# Real-time numerical forecast of global epidemic spreading: case study of 2009 A/H1N1pdm

**DOI:** 10.1186/1741-7015-10-165

**Published:** 2012-12-13

**Authors:** Michele Tizzoni, Paolo Bajardi, Chiara Poletto, José J Ramasco, Duygu Balcan, Bruno Gonçalves, Nicola Perra, Vittoria Colizza, Alessandro Vespignani

**Affiliations:** 1 Computational Epidemiology Laboratory, Institute for Scientific Interchange (ISI), Torino, Italy; 2 Department of Veterinary Sciences, University of Torino, Italy; 3 INSERM, U707, Paris, France; 4 Instituto de Física Interdisciplinar y Sistemas Complejos IFISC (CSIC-UIB), Palma de Mallorca, Spain; 5 Centre de Physique Théorique (CNRS UMR 6207), Marseille, France; 6 Department of Health Sciences and College of Computer and Information Sciences, Northeastern University, Boston MA 02115 USA; 7 UPMC Université Paris 06, Faculté de Médecine Pierre et Marie Curie, UMR S 707, Paris, France; 8 Institute for Scientific Interchange (ISI), Torino, Italy; 9 Institute for Quantitative Social Sciences at Harvard University, Cambridge MA, 02138 USA

**Keywords:** computational epidemiology, H1N1 influenza pandemic, prediction, validation.

## Abstract

**Background:**

Mathematical and computational models for infectious diseases are increasingly used to support public-health decisions; however, their reliability is currently under debate. Real-time forecasts of epidemic spread using data-driven models have been hindered by the technical challenges posed by parameter estimation and validation. Data gathered for the 2009 H1N1 influenza crisis represent an unprecedented opportunity to validate real-time model predictions and define the main success criteria for different approaches.

**Methods:**

We used the Global Epidemic and Mobility Model to generate stochastic simulations of epidemic spread worldwide, yielding (among other measures) the incidence and seeding events at a daily resolution for 3,362 subpopulations in 220 countries. Using a Monte Carlo Maximum Likelihood analysis, the model provided an estimate of the seasonal transmission potential during the early phase of the H1N1 pandemic and generated ensemble forecasts for the activity peaks in the northern hemisphere in the fall/winter wave. These results were validated against the real-life surveillance data collected in 48 countries, and their robustness assessed by focusing on 1) the peak timing of the pandemic; 2) the level of spatial resolution allowed by the model; and 3) the clinical attack rate and the effectiveness of the vaccine. In addition, we studied the effect of data incompleteness on the prediction reliability.

**Results:**

Real-time predictions of the peak timing are found to be in good agreement with the empirical data, showing strong robustness to data that may not be accessible in real time (such as pre-exposure immunity and adherence to vaccination campaigns), but that affect the predictions for the attack rates. The timing and spatial unfolding of the pandemic are critically sensitive to the level of mobility data integrated into the model.

**Conclusions:**

Our results show that large-scale models can be used to provide valuable real-time forecasts of influenza spreading, but they require high-performance computing. The quality of the forecast depends on the level of data integration, thus stressing the need for high-quality data in population-based models, and of progressive updates of validated available empirical knowledge to inform these models.

## Background

Over the past 10 years, the real-world accuracy of mathematical and computational models (MCMs) used in epidemiology has been considerably improved by the integration of large-scale datasets and explicit simulations of entire populations down to the scale of single individuals [1-9]. MCMs have gained in importance in the public-health domain, especially in infectious disease epidemiology, by providing rationales and quantitative analysis to support decision-making and policy-making processes [5,6,10-15]. Although there are contrasting opinions among modelers about the value of MCMs in epidemiology [16], many researchers advocate the use of these models as predictive tools [17].

With regard to modeling, it is important to distinguish between two different types of predictions [18]. The first class of predictions, or projections, offered by models is the classic scenario and 'what if' analysis. In this case, prototypical values for the basic disease parameters and other key parameters, such as time of implementation of specific policies, are assumed in the MCM to produce plausible scenarios for the epidemics and to evaluate containment/mitigation procedures as a function of the explored parameter space. Over the past few years, a large body of work has been published, aimed at informing contingency plans for pandemic preparedness [19-24].

A more difficult challenge compared with scenario analysis is the use of MCMs for the real-time forecasting of unfolding epidemics. It must also be said that forecasting approaches contain a number of assumptions, such as those introduced by the model structure, scale, and implementation techniques. However, in forecasting approaches, the model has to be calibrated by using statistical estimates based on the analysis of epidemic outbreak data for as many key parameters as possible, and possibly by matching less crucial parameters with published historical data. One major technical problem for real-time forecasting is that some parameters, such as the basic reproduction number, are not absolute quantities, and are very dependent on the choice of the model and model parameterization. Two models with different assumptions may reproduce an epidemic profile equally well by using slightly different values of the basic reproduction number, because of the different modeling assumptions used [25]. Thus, within each modeling framework, it is important to have techniques for parameter estimation that are self-consistent with the model assumptions, and cannot be generally imported from other studies. In data-driven MCMs, the self-consistent calibration of the model represents a real challenge because of the number of estimated parameters and the computational costs needed in the case of stochastic individual-based models. Finally, another major problem hindering the advance of real-time forecasting with MCMs is model validation. Real-time forecasting has to be validated using datasets that are independent from those used for the model calibration. Only a few events in recent times have offered the possibility of *a posteriori *validation of the real-time forecasting of MCMs, using rich and high-quality datasets [26].

The 2009 H1N1 influenza pandemic indicated an important role for MCMs in the real-time analysis of disease dynamics and propagation [2,27-39]. Given the uncertainty associated with the emergence of a new virus, such models allowed estimation of unknown epidemiological parameters, description of the observed epidemic propagation, interpretation of surveillance data, exploration of possible scenarios, estimation of the efficacy of intervention measures, and predictions of future influenza activity. The data gathered during the course of the pandemic can now be used to compare with the estimates calculated by the models, and thus these represent an unprecedented opportunity to validate and assess the results obtained by MCM approaches.

In this study, we assessed results obtained using the Global Epidemic and Mobility (GLEAM) computational model [2,3]. This model integrates high-resolution data on human demography and mobility on a worldwide scale in a metapopulation stochastic epidemic framework. With the emergence of the novel H1N1 virus in 2009, the model offered the opportunity to study the spread of the pandemic in real time, and thus evaluate specific public-health actions and provide stochastic forecasts of its future unfolding. The basic model parameters (transmissibility and seasonality) were obtained with a Monte Carlo Maximum Likelihood (MCML)-based approach using the chronological data on the pandemic invasion up to 18 June 2009 [2]. This procedure, although extremely costly in terms of computational time (more than 10^6 ^simulations were generated), can be performed in real time using a supercomputer. The obtained estimates were used to generate a large number of nominally identically initialized numerical stochastic simulations of the global progression of the H1N1 pandemic after 18 June 2009. The simulations provide, for each point in space and time allowed by the resolution of the model, the set of possible epidemic evolution by statistically defining the median, mean, and reference range of a number of epidemic parameters, including newly generated cases, seeding events, and time of arrival of the infection. For the model, we used 3,362 subpopulations in 220 countries worldwide, with a geographical resolution of 15 × 15 minutes of arc, and the time scale of a single day. Based on the early data of the H1N1 pandemic up to June 2009, the model allowed the stochastic forecasting of the activity peak of the fall/winter wave in the northern hemisphere, along with other quantities of interest. The forecasts were published in September 2009 [2], well before the peak weeks of epidemic activity in the northern hemisphere.

The aim of this study was to validate the model's predictions by comparing them with real-life data collected from surveillance and virologic sources in 48 countries in the northern hemisphere during the course of the pandemic. These data allowed independent validation of the obtained results and also allowed the accuracy of the model to be tested. Specifically, we considered the validity of the predicted peak time of the fall wave in the northern hemisphere, the clinical attack rate, and the effectiveness of vaccination. Furthermore, we analyzed results at a finer spatial resolution to ascertain the validity of the model on scales smaller than country level. Using the surveillance data, the timing of the pandemic activity peak was found to fall within the prediction interval for 87% of the countries. In the 13% of the cases falling outside the 95% reference range, the offset with respect to the confidence interval was, at most, 2 weeks at the country level. Because the activity peak in each country is defined as an average over regions composed of many different subpopulations, where data were available we have provided the analysis broken down into smaller surveillance regions, obtaining very good agreement between the model results and data. We also integrated into the model all available data on the vaccination campaigns in 27 countries, and compared the predicted incidence intervals with official estimates such as those produced by the Center for Disease Control and Prevention in the USA. In addition, we analyzed the effect of introducing into the model predictions a number of additional factors that were only known at the end of the pandemic, such as pre-existing immunity, and found that the epidemic timing results were sufficiently robust to cope with changes in these parameters.

Finally, we explored the robustness of the stochastic forecast as a function of the completeness of the data integrated into the model. In particular, one subject of debate has been the level of detail about the international aviation transportation network that would be required to reliably simulate the spreading of infectious diseases worldwide. Whereas the GLEAM model used the full international aviation database, many previous studies have focused only on partial datasets that comprise the top 30% or less of the full dataset [4,6,9,14,29,40,41]. We show that working with partial datasets considerably reduces the accuracy of the predictions at both the local and the global level.

This study shows that although supercomputing capabilities are required, data-driven MCM allows real-time forecasting of emerging influenza-like illnesses (ILIs) with an accuracy that can provide valuable information to inform public-health decision-making. The GLEAM computational tool also allows the introduction of further details in the population structure, such as age classes, and it has been aligned with an agent-based model [42], thus providing avenues for the development of hybrid computational approaches that are able to use different levels of data integration in different subpopulations, with an appropriate compromise between computational requirements and resolution scale of the results.

## Methods

### Model

We used a data-driven global stochastic epidemic model, which is based on the metapopulation approach [4-6,9,14,22,43-48]. The model has been extensively described previously, and all the technical details and the algorithms underpinning the model results reported [2,3,49]. By integrating real demographic and mobility data, the model divides the world population into geographic census areas that are defined around transportation hubs and connected by mobility fluxes, which then defines a subpopulation network. Within each subpopulation, a compartmental structure models the disease spread between individuals. Individuals can move from one subpopulation to another along the mobility network; in this way, an outbreak originating in a seed subpopulation can lead to a global-scale epidemic. The GLEAM model can simulate the global spread of ILIs, and also allows study of the implementation of a wide range of intervention strategies, including vaccinations, antiviral treatment, and travel restrictions (which can be temporally and geographically dependent), to model the different measures adopted by countries in response to an ongoing pandemic. The GLEAM model architecture integrates three different data layers: 1) the population layer, 2) the transportation mobility layer, and 3) the epidemic layer.

The population layer is based on the high-resolution population database of the 'Gridded Population of the World' project of the Socioeconomic Data and Application Center at Columbia University (SEDAC) [50]. This database provides a population estimate by using a grid of cells covering the whole planet, with a resolution of 15 × 15 minutes of arc. The subpopulations of the metapopulation structure correspond to geographic census areas defined around transportation hubs, which are represented by the world airports, as provided by international databases of air travel. The census areas are obtained using a Voronoi-like tessellation of the Earth's surface by assigning each cell of the grid to the closest airport, taking into account distance constraints [3]. The resulting network of subpopulations counts 3,362 census areas in 220 different countries.

The mobility layer takes into account the multiscale nature of human mobility. The GLEAM model integrates the mobility by global air travel (obtained from the International Air Transport Association [51] and Official Airline Guide [52] databases) and the short-scale mobility between adjacent subpopulations, which represents the daily commuting patterns of individuals. We obtained the commuting fluxes by collecting and integrating the data of 30 countries in 5 continents across the world [3] (see Additional file 1A). The model simulates the number of passengers traveling daily worldwide by using the real data obtained from the airline transportation databases, which contain the number of available seats on each airline connection in the world. The commuting short-range couplings between subpopulations are accounted for by defining the effective force of infections in subpopulations connected by commuting flows [3,53,54].

**Figure 1:**
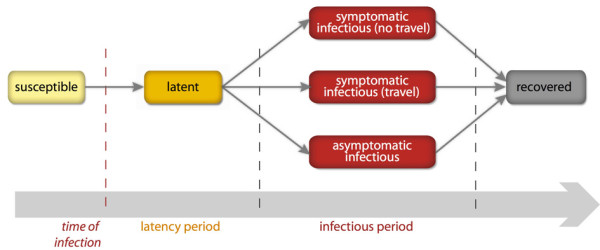
**Natural history of influenza**. After acquiring the infection, a susceptible individual enters the latent compartment, where he is infected but not yet infectious. After the average latency period, each infected individual becomes infectious, and may or may not show symptoms. Symptomatic cases are more infectious than asymptomatic cases. Finally, all infected individuals recover after the average infectious period and become immune to the disease.

The epidemic model within each subpopulation considers a compartmental approach specific for the disease under study [55]. In the present application to the H1N1 pandemic, we assumed that each individual can be in one of the following discrete states: susceptible, latent, symptomatic infectious able to travel, symptomatic infectious unable to travel, asymptomatic infectious, and permanently recovered [2]. The model assumes homogeneous mixing within each subpopulation. The disease transmission rate of symptomatic infectious individuals is *β*, and it is assumed to be rescaled by a factor *r_β _*= 50% for asymptomatic individuals [24,27]. After the infection, susceptible individuals enter the latent compartment, where they are infected but not yet contagious. After the latency period, assumed to be equal to the incubation period and of average duration *ε^-1^*, exposed individuals become infectious and have a probability (1-*p_a_*) of developing clinical symptoms, with *p_a _*considered to be the probability of becoming asymptomatic equal to 33% [56]. Change in traveling behavior after the onset of symptoms is modeled by setting to 50% the probability 1-*p*_t _that individuals would not travel when ill [24]. Eventually, infected individuals recover after the average infectious period *μ*^-*1*^, and they are no longer susceptible. Figure 1 shows the natural history of influenza simulated using the H1N1 GLEAM model. All the stochastic processes modeling the transitions of individuals in the different compartments and their mobility are mathematically defined by discrete stochastic chain binomial and multinomial processes [57,58] to preserve the discrete and stochastic nature of the processes. Individuals are discrete but indistinguishable, because no additional population structure (for example, households or workplaces) is being considered.

The spreading rate of the disease at the level of a single subpopulation is governed by the basic reproduction number, *R*_0_, which is a function of the parameters defining the natural history of the disease [53]. In our study, the basic reproduction number can be expressed as:

$$R_{0}=\frac{\beta }{\mu }\left[r_{\beta }p_{a}+\left(1-p_{a}\right)\right].$$

However, in a metapopulation framework in which space is explicitly considered, the reproductive number is dependent on space and time, and it is more appropriate to define an effective reproduction number *R*(*t*). In more detail, to take into account seasonal effects in the transmission of influenza, we considered a seasonal forcing of the reproduction number, dependent on the calendar time and the region considered. We assumed the world is divided into three regions, delimited by the two Tropics: the northern hemisphere, the southern hemisphere, and the tropical region. We denoted by *R*_0 _the reference value of the reproduction number in the Tropics that needed to be estimated from empirical data of the epidemic. The model reproduces seasonality by means of a sinusoidal rescaling of *R*_0_, by a factor ranging from *α*_min _(during the summer season) to *α*_max _(during the winter season) [9].

The rescaling function has the form:

$$\alpha \left(t\right)=\frac{1}{2}\left[\left(\alpha_{\text{max}}-\alpha_{\text{min}}\right)\text{sin}\left(\frac{2\pi }{365}\left(t-t_{\text{max}}\right)+\frac{\pi }{2}\right)+\alpha_{\text{max}}+\alpha_{\text{min}}\right],$$

where the time of year for the minimum and maximum is fixed and is based on historical data and previous models set at 15 July and 15 January, in opposition in the northern and southern hemispheres. Values of *α*_min _are typically very small for seasonal influenza, so that during the summer season, the effective reproduction number is rescaled to values of less than 1, which are below the threshold for transmission. We set *α*_max _to 1.1, corresponding to a mild increase in disease transmissibility during winter compared with the reference value. Thus, *α*_min _is one of the key parameters to be calibrated from the empirical data of the initial invasion of H1N1 pandemic in order to assess the seasonal variation of the transmissibility of the pandemic virus (see the subsection entitled 'Monte Carlo Maximum Likelihood parameter estimate').

### Computational implementation

**Figure 2:**
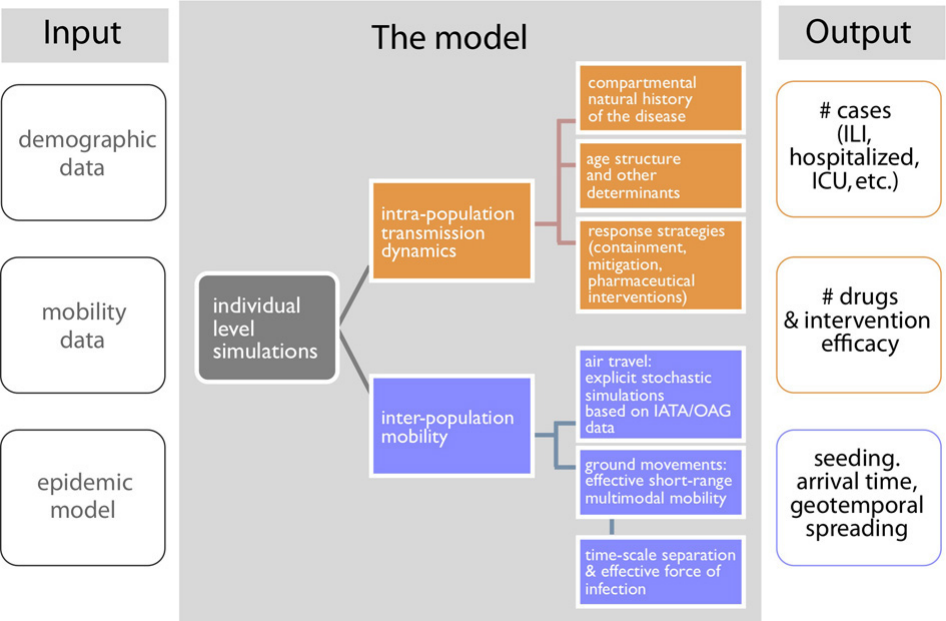
**Schematic illustration of the model flowchart**. The Global Epidemic and Mobility (GLEAM) computational model is based on a data-driven approach. The left column represents the three input databases; the center column represents the dynamic processes that are modeled at each time step, along with their determinants; and the right column indicates example quantities for the model output. Each box is color-coded according to the corresponding dynamic process.

The GLEAM model is implemented in C/C++. A detailed description of the algorithmic structure of GLEAM has been reported previously [49]. Briefly, GLEAM is implemented in a modular manner, with each module performing a specific function. The compartmental model and the epidemic parameters are defined in a configuration text file that is loaded when the program starts. Subsequently, the program loads three data input files: the population database, the short-range mobility network, and the long-range mobility network. During each time step, which represents a full day, the following modules are called into the sequence: air travel, the compartmental transitions (where the force of infection takes into account both the infection dynamics and the short-range movement of individuals), and the partial aggregation of the results at the desired level of geographic resolution. After the last time step, the program generates the final output, which can be further processed for analysis. A schematic representation is provided in Figure 2.

The average running time depends on the number of simulated stochastic realizations of the model and the number of transitions between the epidemic compartments to be modeled. In a cluster with 20 central processing units (Xeon 2 Ghz; Intel Corp., Santa Clara, CA, USA), simulating 2,000 realizations of the model with 365 time steps takes between 3 and 5 hours, depending on the complexity of the compartmentalization and the simulated interventions. Simulating one full pandemic scenario, corresponding to 2,000 realizations for three different seasonality values, on the same cluster took an average of 12 hours.

In July 2010, we released the GLEAMviz Simulator, a software system based on GLEAM, which is publicly available [59], and is based on a client-server system. The GLEAMviz client provides access to all the basic GLEAM features through an interactive graphical user interface. The user can configure freely the compartmental model and simulation scenario by setting compartment-specific variables, transitions, and initial conditions. The user's settings are sent to the GLEAMviz server, which performs the simulations and sends back the analyzed results to the user's client application. Finally, the user can export the results or visualize them in terms of dynamic maps and charts. A full description of the software has been reported previously [60].

### Monte Carlo Maximum Likelihood parameter estimate

In a forecasting approach, the parameters of the model are determined from early data about the epidemic outbreak. Given the number of parameters that a realistic ILI model contains, a full calibration using early data is made difficult by the lack of timely data on the number of cases, the temporal description of the disease in hosts (for example, incubation period, infectious period), the presence of asymptomatic infections, and the rate of transmissibility of these asymptomatic infections. However, for this study, it was possible to distinguish between two classes of parameters. The first class refers to key parameters, such as the specific transmissibility of the disease, which can be determined from the data. The second class refers to parameters that, from sensitivity analysis, have been found to be less crucial in defining the spatiotemporal pattern of the disease propagation and for which plausible and relatively stable values can generally be found in the literature. In the analysis of the H1N1pdm virus, we therefore considered the virus transmissibility and the amplitude of the seasonal rescaling (not its timing) to be the key parameters to estimate. All the other parameters were set to the values reported in the literature for influenza infections (for example, asymptomatic rate and relative infectiousness [24,56], and vaccine and antiviral efficacies [12,61]), or to those available from the analysis of the early outbreak of the 2009 A/H1N1 pandemic [28,29]. Table 1 provides a detailed summary of the parameters used in the model, together with the sensitivity analysis that was performed previously [2] and the additional exploration provided in the present study. In particular, we explored variations in the probability *p_a _*of an individual being asymptomatic (following the evidence provided by a few studies that showed a less frequent occurrence of symptomatic infections than that seen for seasonal influenza strains [62,63]), and of the minimum and maximum scaling factors, *α*_min _and *α*_max_, of the transmissibility during the summer and winter months.

**Table 1:** Epidemiological parameters.^1^

Parameter	Description	Value^2^	Sensitivity analysis range
*R*_0_	Reference reproduction number in the Tropics		

*α*_min_	Minimal seasonality rescaling		

*G_t_*	Generation time, days^3^	3.6 (2.2 to 5.1)	

*μ*^-1^	Mean infectious period, days^3^	2.5 (1.1 to 4.0)	

*ε^-1^*	Average latency period, days	1.1 days	1.1 to 2.5

*r_β_*	Relative infectiousness of asymptomatic individuals	0.5	0.1 to 0.8

*p_a_*	Probability of becoming an asymptomatic individual	0.33	0.33 and 0.5

*p_t_*	Probability of traveling of a symptomatic individual	0.5	0.4 to 0.6

*β*	Transmission rate	*μ*^-1^*R*_0_/(1-*p_a_*-*r_β_p_t_*)	As calculated from the reference range of *R*_0_

*α*_max_	Maximal seasonality rescaling	1.1	1.0 and 1.1

^1^Estimates from the Monte Carlo Maximum Likelihood analysis for various values of the parameter space explored.

^2^The estimated values of *R*_0 _and *α*_min _for all the stochastic forecast outputs are reported in Table 3.

^3^The *G_t_, μ*^-1 ^intervals were set in the MCML analysis and defined by the range of plausible constrained values sampled in the Monte Carlo approach that satisfied a likelihood ratio test at the 5% level (see text).

For the set of key parameters, we used a two-step process that first estimated the reproductive number *R*_0 _in the Tropics region, where seasonality is assumed not to occur, and then estimated the degree of seasonal damping factor by examining a longer time period for international spread to allow for seasonal variations. As reported previously [2], estimation of the reference value of the reproduction number was performed using an MCML technique based on the early chronology of the H1N1 epidemic. We began the model with initial conditions set near La Gloria (in the state of Veracruz, Mexico) on 18 February 2009, as detailed previously [3,27] and according to official Mexican sources [64]. The arrival time of infected individuals in the countries seeded by Mexico is clearly a combination of the number of cases present in the originating country (Mexico) and the mobility network, both within Mexico and connecting Mexico with other countries. By relying on the explicit modeling of the travel behavior of individuals based on the real data, it was possible to shift the estimation of *R*_0 _from the incidence data in the seed country to the timing of the early invasion pattern, with the aim of reducing the errors induced by possible underestimation of cases by surveillance sources. Indeed, the number of cases reported by the surveillance systems was found to be dramatically underestimated, as a result of underdetection and different sampling techniques, as well as changes in surveillance requirements and capacities over time [27,31,65,66]. For this reason, we opted to use as the calibration dataset the first reported case in countries not yet reached by the epidemic. A similar approach was used by Fraser *et al*. [27], and a full sensitivity analysis on the accuracy of this data for the GLEAM model was performed by Balcan *et al*. [2]. Furthermore, the data on the first case were not limited to the arrival date of the person, but usually included additional information about the date of the onset of symptoms, the travel history of the individual, and the supposed source of infection [2].

**Figure 3:**
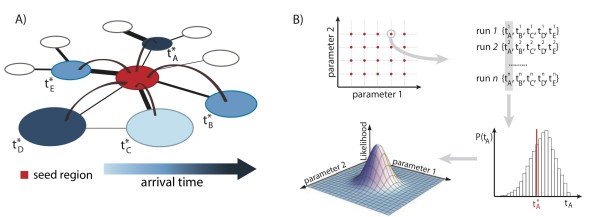
**Monte Carlo Maximum Likelihood (MCML) method used to estimate the transmission potential of the A/H1N1 pandemic**. **(A) **Schematic representation of the invasion dynamics of an emerging infectious disease from the seed subpopulation (red patch) to the neighboring subpopulations connected by means of mobility. The blue color code refers to the arrival time of the first infectious individual. Links of different width represent mobility connections characterized by different mobility flows. **(B) **Flow chart representing the steps that compose the Monte Carlo Maximum Likelihood (MCML) method. First, for each point in the parameter space, we ran 2,000 stochastic realizations, all with the same initial conditions. Second, for each run, we recorded the arrival times in the countries under study. Third, we compared the probability distribution built on the simulated arrival times with the empirically observed arrival times for each country. Finally, we evaluated the likelihood function to find its maximum value, corresponding to the set of parameters that best fits the data.

The MCML analysis is schematically depicted in Figure 3. Being fully stochastic, GLEAM allows for the simulation of a statistical ensemble of epidemic evolutions approximating the probability distribution, associated with an observable statistic that is conditional on the parameters defined as model input. We chose the set of arrival times ({*t*_i_}) for all the countries, *i*, as the statistical observable, and the infection parameters ({*p*}) as the (unknown) input parameters. We then used the simulations to sample the conditional probability distribution in a wide and realistic range of the parameter space. When evaluated in correspondence to the point defined by the empirical dataset ({*t*_i_*}), this probability defines the likelihood function: *L*({*p*}) = *P*({*t*_i_*} | ({*p*}). The simulated arrival times ({*t*_i_}) are in principle statistically dependent variables. Then, to avoid inconvenient correlations in the computation of *L*({*p*}) as a result of multiple possible transmission routes corresponding to the same seeding event, we restricted our analysis to the arrival times of the disease in those countries (n = 12), that were seeded directly by the source (see Additional file 1B). Thus, we used a set of arrival times that were conditionally independent random variables, leading to a factorized expression for the likelihood function. We evaluated the likelihood function by exploring the parameter space using Monte Carlo sampling, and obtained the best estimate for the disease parameters by maximizing the likelihood function in the parameter space. The analysis of the phase space of both transmissibility and generation interval needs particularly intensive computational power. For this reason, we defined the range of acceptable values of the generation interval as those defined by a maximum likelihood test for the ratio of the maximum unconstrained likelihood we could find with the constrained maximum likelihood set at a fixed infectious period. Thus, we excluded values for the infectious period that fell outside the 5% level of the likelihood ratio test. Because of noise and the finite exploration capabilities of the parameter space, all values in the interval of the infectious period within the 5% level were considered as equally possible, and we considered the mean interval value as the reference parameter.

Full calibration of the transmission scenario of the model requires an estimate of the seasonality factor (*α*_min_) to assess the degree of seasonality of the pandemic virus. As previously described for the reproduction number, we based our estimation on the infection arrival times. We used a larger dataset of 93 countries seeded before 18 June, regardless of the origin of the first case, to explore the evolution of the pandemic on a longer time span, over which seasonality effects could be appreciable. Finally, we analyzed the correlation between the empirically observed arrival times and those provided by the model's simulations, by exploring the possible values of *α*_min_. Importantly, we did not fit or calibrate the timing of the seasonal forcing.

Each calibration of the model is computationally expensive, as millions of stochastic simulations are needed to generate the appropriate ensemble describing the spatiotemporal statistical properties that define the likelihood function. Thus, real-time calibrations require supercomputing resources, which in June 2009 were provided to us by the Big Red supercomputer facility [67]. The calibration must be repeated if any new information on the disease is introduced or if specific data that are not initially available will affect the initial conditions or the early phase of the outbreak. Furthermore, each calibration produces different stochastic forecast outputs, as described in the Results section.

### Modeling pandemic management

The GLEAM model allows the implementation of different intervention strategies, including pharmaceutical measures (such as vaccinations and the use of antiviral drugs), and non-pharmaceutical measures (such as travel restrictions and social distancing).

Mass vaccination aims at reducing 1) the susceptibility to infection; 2) the infectiousness if infection occurs; and 3) the probability of developing clinical symptoms [12]. The efficacy of the vaccine with respect to these effects is quantified by the parameters VE_S_, VE_I_, and VE_D_, respectively. We incorporated additional compartments in the disease structure to model mass vaccination [68] (see Additional file 1C), and, based on preliminary studies indicating a similar efficacy to that for the vaccine against seasonal influenza A(H1N1), we referred to previous estimates available in the literature for vaccine efficacy [12], which are similar to those adopted to provide predictions for the H1N1 pandemic [28]. We assumed that a susceptible individual, after vaccination, has a reduced probability of becoming infected by a factor VE_S _= 70%. Then, if infection occurs, his infectiousness is reduced by VE_I _= 30%, while the probability of becoming symptomatic is reduced by VE_D _= 50%. A sensitivity analysis of these values and of the vaccination scheme of the model has been reported previously [68]. The efficacy of vaccines is not instantaneous, and thus we assumed that a single dose of vaccine would be administered, providing protection after a delay of 2 weeks, based on available data from adult clinical studies on the H1N1 influenza vaccine [69]. Given that GLEAM does not consider any additional social or age structure of the population within each geographical census area, the prioritized distribution of vaccines to risk groups cannot be implemented in the model, thus we assumed uniform distribution of the vaccines to a given fraction of the population.

Vaccination is considered in the model in two ways: as a dynamic process simulating the response of countries to the ongoing pandemic based on available data, or as a pre-vaccination scenario. The latter assumes that a fraction of the population of each country (corresponding to 20%, 30%, or 50% of the total) was already vaccinated before the outbreak started in Mexico, that is, at time *t *= 0 of the simulation, simulating a possible universal pre-vaccination to a given fraction of the population before the spread occurs in that region [28], the availability of a pre-prepared vaccine [12,21-23], or a possible degree of pre-existing immunity in the population. The reactive situation, in which countries implement the vaccination campaign after the vaccine has become available, is based on empirical data of the start and end dates of the vaccination campaign, and of the total coverage of the population vaccinated, based on reports provided by national agencies. During the summer of 2009, several countries in the northern hemisphere scheduled vaccination campaigns to mitigate the incoming pandemic winter wave. The simulations reproduced the vaccination campaigns put into action during fall/winter 2009, taking into account the differences between countries in the deployment of vaccines, given the availability of vaccines, and the starting dates for the vaccine stockpile availability. We also assumed that once the national campaign started in a given country, susceptible individuals would be vaccinated daily at a constant rate, calculated on the basis of the campaign's duration, until the desired final coverage is reached. Daily administration rates per country varied from 0.01% to 1.1%, and were explicitly implemented in the model differently from scenario-like approaches, for which this value is generally assumed to be equal to 0.5% [13] or 1% [12,28,29]. Data on observed vaccination campaigns at the country level are presented in Table 2, and the corresponding sources are reported in Additional file 1E.

**Table 2:** Mass vaccination campaigns in the northern hemisphere.^1,2^

Country	Mass vaccination starting date	Final vaccine uptake, %	Daily administration rate, %^3^
China	September 14, 2009	6	0.04

Hungary	October 1, 2009	30	0.24

United States	October 5, 2009	27	0.2

Canada	October 12, 2009	45	0.4

Italy	October 12, 2009	1.5	0.01

Japan	October 19, 2009	17	0.15

Israel	October 19, 2009	9	0.03

France	October 20, 2009	9	0.08

Sweden	October 21, 2009	60	0.35

UK	October 26, 2009	8	0.05

Germany	October 26, 2009	8	0.1

Portugal	October 26, 2009	3	0.04

Finland	October 26, 2009	50	0.4

Austria	October 26, 2009	3.3	0.03

Ireland	October 31, 2009	17	0.1

Denmark	November 2, 2009	6	0.07

Turkey	November 2, 2009	3	0.02

Iceland	November 2, 2009	40	0.35

Belgium	November 2, 2009	7.5	0.07

Slovenia	November 2, 2009	5	0.05

Netherlands	November 9, 2009	25	1.1

Switzerland	November 15, 2009	15	0.2

Spain	November 16, 2009	4.5	0.05

Greece	November 16, 2009	3	0.05

Tunisia	November 16, 2009	2.6	0.05

Czech Republic	November 23, 2009	0.6	0.01

Norway	December 1, 2009	45	0.3

^1^Data on the timing and final population coverage of mass vaccination campaigns in the countries of the northern hemisphere, during the 2009 to 2010 winter season.

^2^Sources of the data are reported in Additional file 1, Table S4.

^3^The daily administration rate per country was calculated by assuming a constant rate during the vaccination period indicated by the data up to the final uptake value reached in each country.

Although the treatment of clinical cases with antiviral drugs (neuraminidase inhibitors) aimed at reducing the severity of the disease and the transmissibility while infectious [5,11,13,21-23,70-72], were not implemented systematically during the A/H1N1 pandemic, we considered this possibility in the model in order to investigate any possible modifications to the overall spatiotemporal pattern of the pandemic. In particular, we studied a scenario assuming the prompt detection of symptomatic cases and the rapid administration of the drug to 30% of the clinical cases who would be treated within the first day from the onset of symptoms [2,5]. Moreover, we assumed the efficacy of the drug in reducing transmission to be equal to 62%, and a reduction of 1 day in the total infectious period, as available from the existing literature [22,23], although the evidence of the efficacy of neuraminidase inhibitors in relation to transmission is still under debate [73]. To guarantee a realistic description of the antiviral distribution, we modeled the drug availability in each country by using actual data, collected from the study of Singer *et al*. [74] and from national agencies. Thus, treatment with antiviral drugs was simulated only for those countries that had drug stockpiles available at the beginning of the pandemic. The intervention at the national level was assumed to start with a delay of 3 days after the appearance of the first symptomatic individual in the country, but not before the international pandemic alert released on 25 April. Administration of the drugs was assumed to occur at a constant rate until depletion of the country's stockpile. Figure S1 (see Additional file 1C) displays the compartmental structure in each subpopulation when pharmaceutical measures were considered.

In addition to pharmaceutical measures, we also considered interventions aimed at limiting the mobility of individuals and applied in terms of travel restrictions. The explicit inclusion of the mobility network in the GLEAM model allowed us to apply modifications to individual mobility by air in a variety of forms to take into account real-life behaviors. More specifically, interruption of specific travel routes or airports for an arbitrary period can be considered in the model, and the traffic flows to and from given locations (for example, the outbreak seed) can be reduced and modulated in time, based on real data. Some countries did in fact adopt travel-related measures in an attempt to contain or slow down the international spread of the A/H1N1 virus. In a few extreme cases, the authorities banned all the flights directed to/from Mexico, in order to prevent infected individuals from crossing international borders. These measures, along with self-imposed travel limitations, contributed to a decline of about 40% in international air traffic to and from Mexico after the international alert, which was slowly reduced after June 2009 and had returned back to normal in about 3 months (see references [75,76] for analysis of the data). Thus, simulations with travel-related measures considered the variation over time of the reduction of traffic flows to and from Mexico, as observed in the data.

Finally, control strategies implemented at the level of social groups of single individuals, such as social distancing measures or school closure, can be introduced into the model only by an effective rescaling of the reproduction number for a given time period and in a specific geographic region. Applying this approach, we simulated the interventions that took place in Mexico starting on 24 April and ending on 10 May, using a time-dependent modification of the reproductive number in the country as reported previously [77]. During the period when social distancing was effective in Mexico, we assumed that the reproductive number in Mexico, *R*_Mex_, changed its value to *R*_Mex _= 0.9, resulting in about a 50% reduction from the reference value. Different reduction rates of *R*_Mex _have been tested previously [2], and no significant changes were found.

### Modeling population immunity profile

Taking into account the recent results of serological analyses [78-81], we also considered simulations in which the population had an initial degree of immunity before exposure, and assessed the change in the predictions provided by the model when full susceptibility in the population is taken into account. Measurements of antibodies in serum samples can be used to identify cross-reactivity between antibodies elicited by seasonal influenza viruses circulating before the pandemic, thus providing estimates of pre-exposure immunity to the H1N1 pandemic virus in the population [17,82]. Serological surveys reported evidence of substantial pre-exposure immunity to the 2009 A/H1N1 pandemic virus among older sections of the population. The detected levels of pre-exposure cross-reactive antibodies ranged from 23% of individuals aged 65 years or over in the UK [78], to 30% and 34% for those born before 1950 (that is, those aged 60 years or over in 2009) in Finland [79] and the USA [80], respectively, and 37% in Germany for the same age group [81]. To explore the effects of pre-exposure immunity, we assumed that 33% of individuals older than 60 years would be immune and completely protected against the H1N1 pandemic virus. We used the data from the International Database of the US Census Bureau [83] to estimate the corresponding fraction of each country's total population with pre-exposure immunity, relying on the different national age profiles, given that in this work we did not consider age structure in the GLEAM modeling of the population.

### Surveillance data

To compare our numerical results with the observed temporal and geographic pattern of the pandemic fall/winter wave, we collected data from the monitoring systems of 48 countries in the northern hemisphere, accessing their official websites on a regular weekly basis, and also downloading their final reports at the end of the wave with an assessment of the influenza activity using the most relevant indicators (for the full list of our data sources, see Additional file 1E).

Surveillance systems in the countries under study use different operative methods and a wide range of influenza case definitions to monitor influenza activity within the country. Our data sources reported at least one or more of the following indicators on a weekly basis: ILI incidence, acute respiratory infection (ARI) incidence, fraction of ILI visits or fraction of ILI patients per sentinel doctor, and number of H1N1pdm laboratory-confirmed cases. All of the indicators are generally based on the number of individuals that seek health care and who have respiratory symptoms that can be specifically diagnosed as ILI or, with a broader set of possible causes, as ARIs. Specific virologic analyses are typically conducted on a subset of patients to monitor the activity per strain. Worldwide surveillance networks adopt a large variety of clinical case definitions, and there is currently no international consensus on a 'gold standard' for the case definition of influenza (see, for instance, the UK Health Protection Agency [84] and the US Centers for Disease Control [85] definitions for H1N1 cases). Nevertheless, most surveillance networks share common symptoms or common generic terms in their definitions [86], and both the ILI and ARI case definitions were found to be good indicators of influenza activity in Europe [87]. The harmonization of influenza monitoring across countries with a single ILI case definition is currently being tested in Europe, using online surveillance systems [88].

Depending on the system, indicators may need to be adjusted to specific normalization factors or consultation rates to extrapolate numbers of cases with respect to the whole population. In particular, healthcare-seeking behavior is a parameter that is difficult to estimate. Moreover, it may vary across age groups and it may also change over time, especially during a health emergency such as a pandemic influenza, as a result of government advice, media coverage, and resulting public anxiety [89]. Given that we were interested in the fall/winter wave of the 2009 H1N1 pandemic, we assumed a constant surveillance effort and consultation rate across time in the countries under study (the same assumption would not be true when comparing the first and the second waves, as, for example, in the UK [89-91]). In addition, because surveillance data were used in this work only to provide a comparison with the timing of our predictions, we disregarded normalization factors and consultation rates, and assumed that surveillance data provided a reliable estimate of the timing of the influenza activity peak using the various available indicators [87]. Finally, to account for the uncertainty intrinsic to empirical data, we used a color gradient to indicate the observed peak weeks, with the limits corresponding to the time interval in which an incidence of greater than 80% of the maximum was observed.

## Results and Discussion

### Stochastic forecast output sets

As discussed in the Methods section, the GLEAM model generates a large number of nominally identically initialized numerical stochastic simulations of an epidemic's global progression. The simulations provide, for each point in space and time as given by the resolution of the model, an ensemble of possible epidemic evolutions. It gives median, mean, and reference ranges for epidemic observables, such as newly generated cases, seeding events, time of arrival of the infection, and number of drugs used. The ensemble forecast and the statistical quantities depend on the key parameters determined by the MCML calibration of the model. Each calibration thus defines a different stochastic forecast output (SFO) set that can be validated against real data.

Each MCML calibration and the corresponding SFO set corresponded to the numerical generation of more than 10^6 ^global simulations, and the manipulation and storage of about 1 terabyte of data.

We considered the following SFO sets and their corresponding calibrations:

• Baseline SFO set. This is the set corresponding to the numerical analysis presented previously [2]. This set was generated in June 2009, and owing to lack of data, it did not include change in traveling behavior and/or pre-exposure immunity. This set was particularly relevant in the validation process because it is the SFO achieved in real time before the unfolding of the winter wave of influenza in the northern hemisphere.

• Reference SFO set. This SFO set was obtained by a calibration that considered the observed drop in travel flow during the early stage of the outbreak, as reported by the Mexican authorities [76]. These data became available in December 2009, and for this reason, could not be considered in our initial work. The reference SFO was then coupled with a series of intervention options (outside Mexico), considered one at a time (described in the Methods section), to assess the effect of the following: travel restrictions of increased magnitude; vaccination campaigns as deployed in reality (obtained from data available after the pandemic); and antiviral treatment and pre-vaccination as hypothetical scenarios. Those interventions, which were implemented well after the start of the pandemic, did not affect the model calibration.

• Pre-exposure immunity SFO set. Based on the recent results of serological analyses [78-81], this set assumed that a fraction of the total population of each country would have pre-exposure immunity to the pandemic virus. This fraction was calculated by relying on the different national age profiles to match the observed pre-exposure immunity in individuals older than 60 years (see Methods). Unlike the other SFO sets, in which interventions starting at a later stage of the epidemic were considered, the pre-exposure immunity SFO set requires performance of a full MCML calibration, given that the initial conditions of the population's immunity profile have changed. The pre-exposure immunity SFO set is also analyzed by including vaccination campaigns.

A schematic description of the components of each SFO set and the interventions considered is provided in Table 3.

**Table 3:** Summary of the A/H1N1pdm Monte Carlo Maximum Likelihood (MCML) calibrations and best parameter estimates.

MCML and interventions^1,2^			
	**Baseline SFO**	**Reference SFO**	**Pre-exposure immunity SFO**

Social distancing in Mexico, April 24-May 10, 2009	✓	✓	✓

Traffic reduction after April 25, 2009		✓	✓

Pre-exposure immunity			✓

Vaccinations campaigns (data-driven)		+	+

Antiviral treatment (hypothetical scenario)		+	

Pre-vaccination (hypothetical scenario)		+	

**MCML estimates^3^**			

Minimal seasonal rescaling factor, *α*_min_^4^	0.65 (0.60 - 0.70)	0.65 (0.60 - 0.70)	0.70 (0.65 - 0.75)

Reference reproduction number in the Tropics, *R*_0_^5^	1.75 (1.64 - 1.88)	1.75 (1.64 - 1.88)	1.8 (1.69 - 1.91)

^1^The '✓' symbol indicates the components considered in each stochastic forecast output (SFO) set and hence also in the calibration.

^2^The + symbol indicates the data-driven interventions and/or hypothetical scenarios considered in addition to each SFO set.

^3^Each MCML calibration and the corresponding set of best estimates was used to generate the stochastic forecast output datasets consisting of an ensemble of possible epidemic evolutions defining a median, mean, and reference range for epidemic observables.

^4^The *α_min _*interval is the best-fit range within the minimal resolution allowed by the Monte Carlo sampling.

^5^For *R*_0_, we report the 95% confidence interval (in square brackets).

Finally, we explored additional SFO sets to perform a sensitivity analysis of the conditions and assumptions considered in the simulations, in which we assessed the role of the following: consideration of a sample of the airline transportation network; the winter and summer rescaling values of the seasonal sinusoidal function (*α*_max _and *α*_min_, respectively); the parameters related to asymptomatic infections; and the initial geographic conditions of the seed outbreak location in Mexico. In all of these cases, a new estimate of the seasonal transmission scenario was performed because the initial conditions had changed. All of the SFO sets explored the evolution of the pandemic over a time span of 1 year, and the results shown in the following sections were obtained from at least 2,000 stochastic simulations.

We report in Table 3 the values of the parameters obtained by the MCML estimate for each of the SFO sets. Generally, the parameter values were not particularly sensitive to the progressive integration of data on reduction of travel to and from Mexico. A rationale for this result is provided in the next section. The values obtained in the different MCML estimates for *R*_0 _ranged from 1.64 to 1.91. It should be noted that this number refers to the reference value, and the effective reproduction number is determined at each time step of the simulation by considering the seasonal effects. This seasonal scaling provides an effective reproduction number in the northern hemisphere, ranging from 1.05 to 1.5 in the spring/summer months, in agreement with published estimates of the reproduction number [27,28]. The time dependence of the seasonally effective reproduction number *R*(*t*) in the northern and southern hemispheres, for the estimated values of *R*_0 _and *α*_min _in the A/H1N1 pandemic baseline SFO set, was calculated (see Additional file 1D, Figure S2).

### Early stage, first-case importations, and travel restrictions

In the early stage of the A/H1N1 2009 pandemic, the worldwide air-transportation network was the main dissemination mechanism from Mexico to the rest of the world. We first assessed the role of the observed travel decline on the MCML estimates and the SFO set for the early stage of the epidemic by using the data on travel to and from Mexico that became available at the end of 2009. We compared the results of the baseline SFO with those of the reference SFO in which the observed travel decline was considered. The discrete stochastic structure of the model allowed tracking of the arrival of each detectable (that is, symptomatic) and non-detectable (that is, latent or asymptomatic) infected individual in any given country. By defining the arrival time as the date on which the first symptomatic case arrived in a given country, it was possible to quantify the delay in the spreading of the epidemic from country to country that was achieved by traffic reduction. The decline of 40% in the travel flows to and from Mexico reported for the month of May 2009 (which was then followed by lowered reductions until a return to normality 3 months later) led to an average delay in the importation of the first case in seeded countries of less than 3 days [69], without altering the MCML estimate of the seasonal transmission. This is consistent with the results we previously obtained in a sensitivity analysis investigating the robustness of the estimation procedure to variations in the chronological data of the first imported case, assuming possible inaccuracies in the reporting [2].

Furthermore, the numerical simulations allowed us to test whether a decrease in travel flows of magnitudes larger than the observed 40% would have provided any additional benefit in slowing down the propagation of the A/H1N1 virus across the world. We considered reductions ranging from 50% to 90% in the air travel flows connecting Mexico with the rest of the world, starting on 25 April, after the international alert, and optimistically assumed prompt implementation of the intervention by the authorities, with no further delays. We also assumed that the reduction would be kept constant across time and would never reduce nor return to normality, which is different from the situation revealed by the real data.

**Figure 4:**
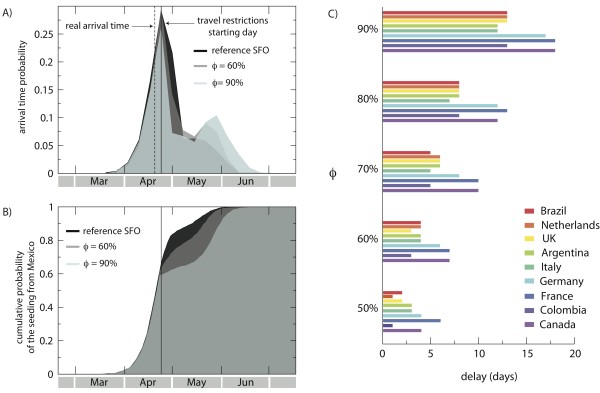
**Travel-related measures in the early stage of the epidemic**. **(A) **Probability distribution of the arrival time (date of arrival of the first symptomatic case) in Germany for different values of traffic reduction, *ϕ*. The vertical dotted line indicates the observed arrival time in the country, as obtained from official reports, and the vertical solid line indicates the starting date of the travel restrictions (25 April, 2009), which was the day after the international alert. The probability distributions were obtained from 2,000 stochastic realizations, and data were binned over 7 days. **(B) **Cumulative probability distributions of the first seeding event from Mexico to Germany for different values of traffic reduction *ϕ*. We considered any source of infection in the seeding event, including symptomatic cases and non-detectable infected cases, such as latent and asymptomatic. **(C) **Delay in the case importation from Mexico to a given country compared with the reference stochastic forecast output (SFO) as a function of the travel reduction *ϕ*. The delay was measured in terms of the date at which the cumulative distribution of the seeding from Mexico (B) reached 90%.

Instead of measuring the average delay only, we considered for every country the probability distribution of the arrival time of the first symptomatic infectious individual, with no regard to the source of infection, which allowed us to take into account the stochasticity of these events in order to explore how the probability distributions would change for increasing travel reductions. Germany is an example where, based on our simulations, the arrival time probability distribution would have peaked a few days later than the real arrival date (Figure 4A). However, travel reductions of a magnitude equal to 60% or 90% would not be able to delay the distribution peak time, and would result only in a change in the tail of the distribution; a more rapid drop after the peak would then be followed by an increase later on, owing to the arrival of cases from countries other than Mexico. By focusing only on the seeding from Mexico, we were able to compute the cumulative distribution of all seeding events, taking into account latent, symptomatic, and asymptomatic infected individuals. We found that the cumulative probability distribution of the seeding could be reduced by travel-related measures, resulting in a slower importation rate (Figure 4B). By fixing the cumulative probability at 90%, we computed the delay induced by the travel reductions for a set of countries (Figure 4C). Even with an unfeasibly large traffic drop of 90%, the achieved delay was less than 20 days. This would offer additional time to activate the pandemic preparedness plans of each country to control the initial local transmission of a novel strain, such as by enhancing surveillance, but it would provide little or no benefit in gaining time for vaccination interventions, given that the scale of vaccine development, production, and distribution is about 6 months.

Confirming previous modeling and theoretical works on travel restrictions in pandemic planning [5,9,12-14,20,92] and empirical studies on entry screening [93], these results suggest that the observed travel drop did not lead to substantial delays in the arrival of the H1N1 epidemic to non-affected areas. In addition, the simulations showed that it would not be possible to contain the pandemic by the sole implementation of travel restrictions, even if these were unfeasibly strict. These results can be rationalized in a theoretical framework characterizing the invasion dynamics of the epidemics at the metapopulation level, and are related to the heterogeneity of the mobility patterns of humans [76,94].

### Pandemic activity peaks in the northern hemisphere

**Figure 5:**
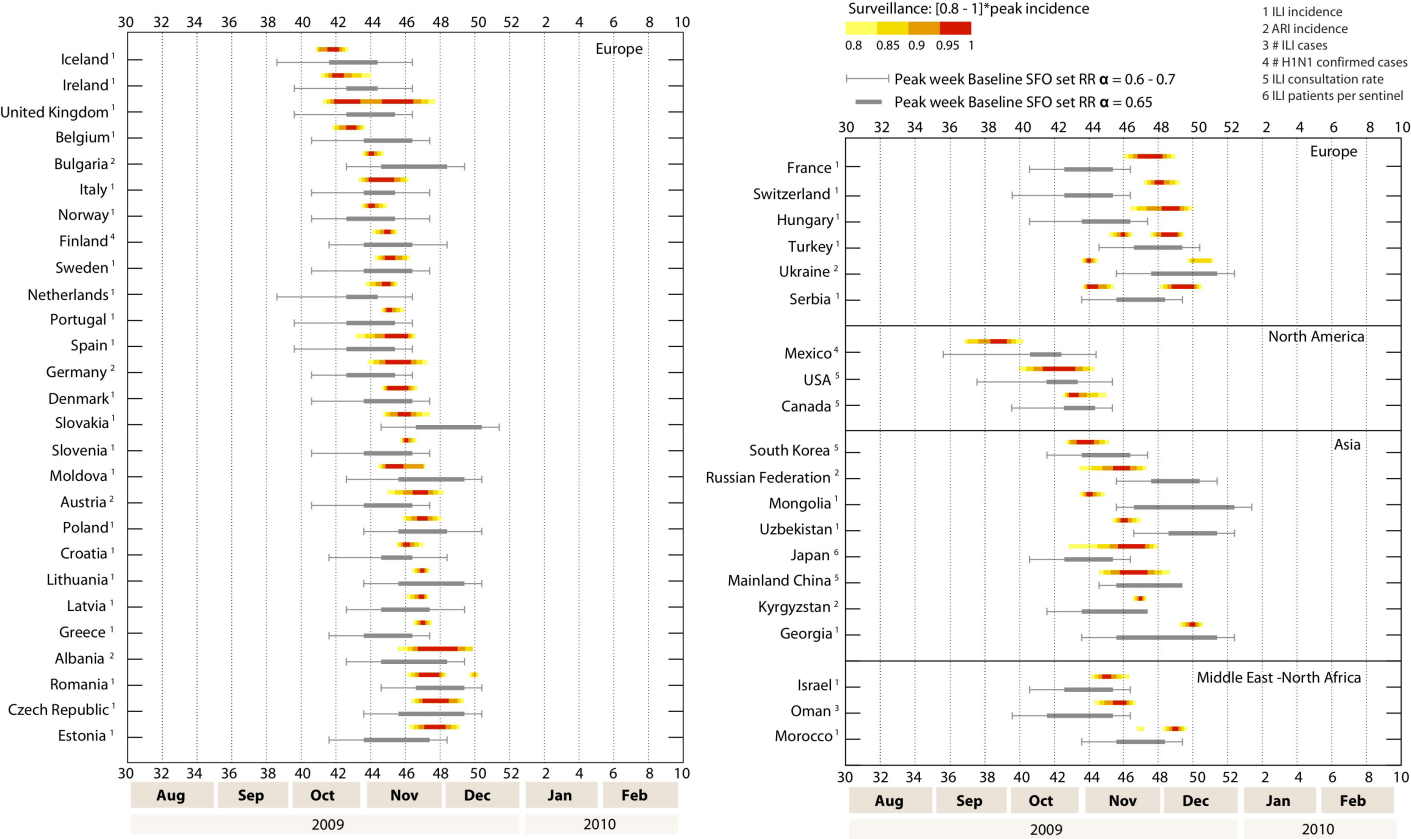
**Peak timing in the northern hemisphere: simulations and real data**. Peak weeks of the epidemic activity in the baseline stochastic forecast output (SFO) (gray). The reference ranges of the simulated peak week were obtained by analysis of 2,000 stochastic realizations of the model for three different values of the seasonal rescaling factor, *α*_min_, of 0.6, 0.65, and 0.7. The peak weeks reported by the surveillance for the fall/winter wave are shown as color gradients, whose limits correspond to the time interval at which an incidence of greater than 80% of the maximum incidence was observed. The numbers 1 to 5 indicate the type of data provided by the surveillance of each country, and the numbered weeks of the year correspond to the calendar used by the US Center for Disease Control and Prevention.

The influenza activity data collected from 48 countries in the northern hemisphere (some of which lie across the northern hemisphere and the Tropics region) showed that most of the countries experienced a single major influenza wave during the fall of 2009. Data from virus specimens collected worldwide indicated that the wave was mainly due to the 2009 A/H1N1 pandemic strain, which was the predominant strain in the 2009 to 2010 season, accounting for more than 90% of the sampled specimens [95]. The influenza activity in these countries peaked during the period October to December, much earlier than the usual timing of seasonal influenza for countries in the northern hemisphere, which generally ranges between January and March. The pandemic peaked first in North America between the end of September and the end of October (in Mexico first, then in the USA, and soon after in Canada), and later in Europe. The situation in Europe was more heterogeneous, leading to an overall range of timing for the week of peak activity from late October to late December. The peak timing from the surveillance data of the various countries was subdivided by world regions and ordered by timing within each region (Figure 5); with few exceptions, the first peak of activity was experienced by countries in Western Europe, later followed by Eastern Europe. Other countries in Asia, the Middle East, and North Africa were also analyzed, showing peak data in the months of November and December.

We found that the peak week correlated significantly with the total air traffic of each individual country to and from North America, both in the data and in the model output (Table 4). Similar correlations were found when we restricted the analysis to European countries, with a correlation between the peak week and the intra-European air traffic of any individual country, which was captured both by the data and the model. The peak week was also found to be positively correlated with a country's longitude, generally indicating a west to east pattern, and this correlation seemed to be stronger in Europe (as reported previously [39]), both in the data and the model output, probably as a result of the large air traffic between Western Europe and North America. A weak correlation was found between the peak week and the latitude of individual countries, both at the global level and when restricting the analysis to European countries.

**Table 4:** Correlation of population variables and epidemic statistics as seen and predicted by the model.

Full dataset					
**Epidemic statistic**	**Population variable**	**Correlation observed in real data^a^**		**Correlation predicted by the model^a^**	
		
		***r***	***P***	***r***	***P***

Worldwide					

Peak week	Air traffic to/from North America	-0.30	0.042	-0.30	0.044

Peak week	Longitude^b^	0.27	0.071	0.42	0.003

Peak week	Latitude^b^	-0.15	0.317	0.02	0.882

Peak week	Vaccine uptake	-0.37	0.069	-0.38	0.060

Attack rate reduction, %	Vaccine uptake			0.73	< 0.001

**European countries only**					

Peak week	Intra-EU air traffic	-0.15	0.391	-0.48	0.003

Peak week	Air traffic to/from North America	-0.26	0.151	-0.37	0.003

Peak week	Longitude^b^	0.54	< 0.001	0.74	< 0.001

Peak week	Latitude^b^	-0.32	0.07	-0.23	0.178

Peak week	Vaccine uptake	-0.29	0.204	-0.28	0.212

Attack rate reduction, %	Vaccine uptake			0.69	< 0.001

^a^Values of the Pearson's correlation coefficient, *r*, along with the corresponding *P *value, were measured between the epidemic statistics and the population variables of the countries appearing in Figure 5.

^b^Longitude and latitude are those of the capital city of the country.

The empirical data were compared with the results of the numerical simulations performed for the baseline SFO set (Figure 5). In light of the results presented in the previous subsection, we checked whether the timing of the simulated epidemic activity showed any differences between the reference SFO set (in which the observed travel drop during the early stage was incorporated into the model) and the baseline SFO set (in which that aspect was not considered because the data were not yet available), for which predictions were reported previously [2]. We found that 95% of the reference range of the simulated peak week was obtained from the minimal seasonality rescaling, *α*_min_, in the range of 0.6 to 0.7, estimated from the calibration. The SFO sets therefore seemed to be in very good agreement with the empirical data, showing that the latter fell within the confidence interval of numerical results in most of the countries under study. Only for 13% of the countries did our predictions differ from the observed timing of the influenza activity, and in these, the early arrival (France, Switzerland, Hungary) or the delay (Ukraine, Mongolia, Uzbekistan) compared with the simulations was 2 weeks at most, measured from peak week to the closest end value of the reference range of the numerical results.

**Figure 6:**
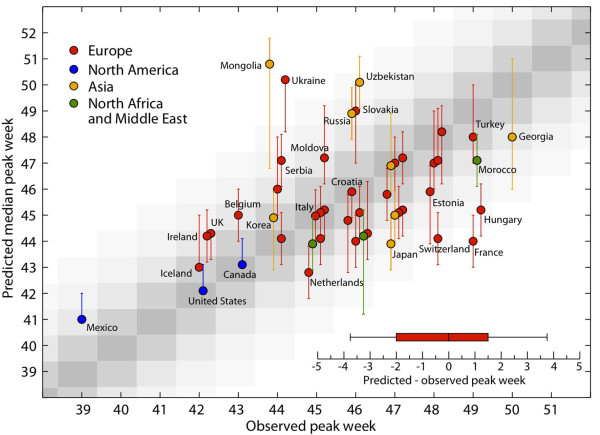
**Statistical association between the predicted and observed activity peaks**. Peak week as simulated by the model in the baseline stochastic forecast output (SFO) set with *α*_min _= 0.65 versus the peak week observed by surveillance systems in the countries outlined in Figure 5. The reference ranges of the simulated peak week were obtained by analysis of 2,000 stochastic realizations of the model. In the inset, we show the box plot indicating the distribution of the differences between the simulated peak week for the baseline SFO set with *α*_min _= 0.65, and the observed peak week.

We compared the predicted peak week for the baseline SFO set with *α*_min _= 0.65 against the observed peak week, and found a range of 4 weeks' difference (gray shaded area in Figure 6) between the observed and the predicted peak week. There was a significant correlation between the data and the prediction (Spearman correlation coefficient 0.48, *P *= 0.0001). The error lay within 4 weeks for 95% of the countries, and within 2 weeks for 50% of them, and the median error was 0 (Figure 6). We also compared the median predicted peak weeks and the observed peak weeks using the Wilcoxon signed-rank test, and found no significant difference between the two sets, at the 0.01 level of significance (*Z *score < 2.33).

It should be noted that the obtained results are highly non-trivial because of the anticipated peak of the pandemic in the northern hemisphere. The GLEAM model does not alter the timing of the seasonal forcing that would intuitively generate an activity peak in mid-January. The anticipated peaks are thus a genuine result originating from the initial condition of the pandemic, the transmissibility estimate, and the spreading pattern generated by the human mobility integrated into the model. In this sense, the offset of 1 or 2 weeks observed for a limited number of countries can still be considered a good result, compared with the several months for dispersion allowed in principle by the seasonal forcing only.

An offset of 2 or 3 weeks for the forecast may be due not only to the model approximations and components but also to other factors that were not considered in the GLEAM model because of lack of data at the time of the predictions or because they would require country-specific implementation in the model. An example is provided by the case of France, where the beginning of the exponential increase of the incidence curve in fall 2009 was interrupted by a sudden drop [96], corresponding to a countrywide school break of 2 weeks (during weeks 43 and 44), consistent with the results observed from the analysis of the timing of holidays and from 21 years of French surveillance data of ILI [97]. The fact that the peak appeared 2 weeks later than predicted by the model may thus be explained by the delaying effect produced by the school holiday. This and other effects, although they could be implemented in the model through explicit or effective means, would require the collection of country-specific data worldwide for a large spectrum of events. Although we performed simulations with explicit travel drops and vaccination campaigns at the country level as they took place in reality (see previous and next subsection), the inclusion of country-specific additional factors, such as school holidays, were beyond the scope of this study.

Calibration of GLEAM based on the chronological data of the H1N1 invasion up to 18 June 2009 was able to provide accurate predictions (2 to 4 months in advance) of the timing of the peak activity in countries in the northern hemisphere (Figure 5, Figure 6). This information provided additional support for the evaluation of real-time interventions aimed at mitigating the pandemic [68], and was made available to public-health policymakers to provide guidance for strategic planning. In addition, the large-scale extent of this approach enabled predictions for countries not usually considered by other modeling approaches that require large detailed datasets to build synthetic populations, and described their behavior at the individual level. Other than the USA [7,13,22,24,98], specific European countries [12,15], or the European continent as a whole [8], other developed countries do not appear in modeling studies, and underdeveloped countries have been considered in agent-based models in only a few cases, such as in pandemic preparedness studies that focused on Thailand with regard to the possible emergence of a pandemic from the H5N1 avian flu virus [11,24].

### Spatial resolution analysis

To test the reliability of the GLEAM model on a smaller geographical scale and in countries with heterogeneous climatic structures, we validated the baseline SFO for two countries, India and Canada, for which there are no specific models available and which are characterized by their large geographical extension. Furthermore, the coupling between the different regions of those countries is complicated by the presence of different seasonal areas within the same country (in the case of India) and by a highly structured population with a large extension of inhabited areas (in the case of Canada). We expected this to have a strong effect on the timing of the pandemic activity peak [9].

**Figure 7:**
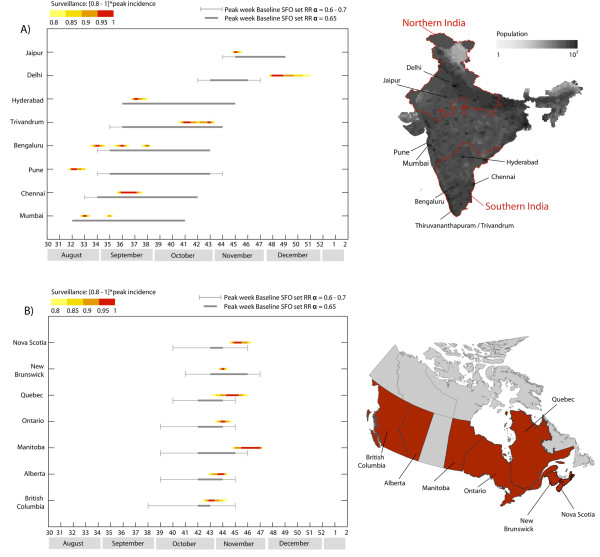
**Peak timing in India and Canada: simulations and real data**. **(A) **Peak weeks of the epidemic activity in the baseline stochastic forecast output (SFO) (gray) for eight Indian cities, ordered by decreasing latitude from top to bottom. Right: map of India, showing the Indian population distribution and the subdivision in North, South, and Central regions. **(B) **Peak weeks of the epidemic activity in the baseline SFO (gray) for seven Canadian provinces, ordered eastward from top to bottom. Right: map of Canada, where the Canadian provinces under study are highlighted in red. The 95% reference ranges of the simulated peak week were obtained by analysis of 2,000 stochastic realizations of the model for three different values of the seasonal rescaling factor, *α*_min _= 0.6, 0.65, and 0.7. The peak weeks reported by the surveillance are shown as color gradients, whose limits correspond to the time interval where an incidence of greater than 80% of the maximum incidence was observed. Both maps were made exclusively for this manuscript and are not subject to copyright.

India is roughly halved by the Tropic of Cancer. Based on information from the Indian surveillance system, we identified three regions in the country: northern, southern, and central India (see map in Figure 7A). Northern India belongs to the northern hemisphere, where the seasonality rescaling function modulates the reproductive number (Figure S2), whereas southern India is a tropical region, where the reproductive number is fixed to its reference value *R*_0_. Central India is crossed by the Tropic of Cancer, and therefore extends into both seasonal regions. Given this subdivision of the country into large regions, we examined in more detail the situation of eight large Indian cities for which influenza surveillance data were available. The pandemic wave peaked first in the cities in central and southern India, between August and October, whereas northern Indian cities experienced the activity peak later, in November and December (Figure 7A). Concerning the reference SFO results, the six cities in central and southern India are characterized by much wider reference ranges than those typically found for cities and countries in the northern hemisphere. This is due to the lack of seasonal forcing, which generally reduces stochastic effects and thus provides a smaller reference range for the SFO datasets. However, the timing reproduced by GLEAM simulations was able to capture the early wave observed in central and southern India, which was then followed by the later peak of activity experienced in the cities of Jaipur and Delhi, which belong to the northern hemisphere. The SFO seems to indicate that the real mobility and population data integrated into the model are sufficient to provide useful information on the timing of the pandemic within the country, although the error bars for the results covered a duration of 4 to 6 weeks. At the national level, the aggregation of the pandemic waves experienced in the different regions at different times resulted in a double peak of the total incidence curve, as reported by the Indian surveillance system. In our reference SFO set, the incidence curve of India presented a double peak in more than 90% of the stochastic realizations of the model, reproducing the same seasonal pattern observed in reality (see Figure S3 for a detailed analysis of the prediction at the national level).

By contrast, Canada falls completely within the northern hemisphere, where the seasonal rescaling function modulates the value of *R*_0_, leading to higher transmissibility rates during wintertime. The Canadian case is of interest because the country has one of the lowest population densities in the world, and is characterized by a largely heterogeneous geographical distribution, with cities mainly scattered along the border with the USA, and varying densities from west to east. Despite the synchronization effect of epidemic waves produced by seasonal rescaling, the heterogeneous population distribution in a vast area leaves room for an important role of the mobility pattern in shaping the timing of the arrival of the epidemic and its peak activity in different regions. We collected the weekly incidence data reported by the surveillance systems of seven Canadian provinces (Alberta, British Columbia, Manitoba, New Brunswick, Nova Scotia, Quebec, and Ontario, which account for more than 94% of the Canadian population), and compared the observed activity peak with the simulated peak in our baseline SFO. The pandemic activity peaked between the end of October and the end of November (weeks 43 to 47), with the timing over all regions spanning an entire month, and with the presence of narrow to broad peaks in the incidence profiles, as shown, for instance, by the cases of New Brunswick and Manitoba, respectively (Figure 7B). The 95% reference ranges of the peak week in our reference SFO simulations were in good agreement with the surveillance data, and were able to reproduce a variation in the timing of the peak occurrence across the country. This is a result of the interplay of the region's connection to the rest of the world where the epidemic was unfolding, and the intra-country connections and population distribution that drove the local epidemic propagation and internal coupling across regions due to local mobility. As expected, those regions that are better connected to the rest of the world through international travel flows of passengers experienced the peak earlier, with the exception of New Brunswick, which synchronized with the early timing of the peak; this may be explained by the large commuting flows from New Brunswick to the neighboring regions [3]. For the sake of completeness, we also provide a validation of the model results for Mexico, with a breakdown by Mexican region (see Additional file 1G).

### The effect of vaccination on peak timing

**Figure 8:**
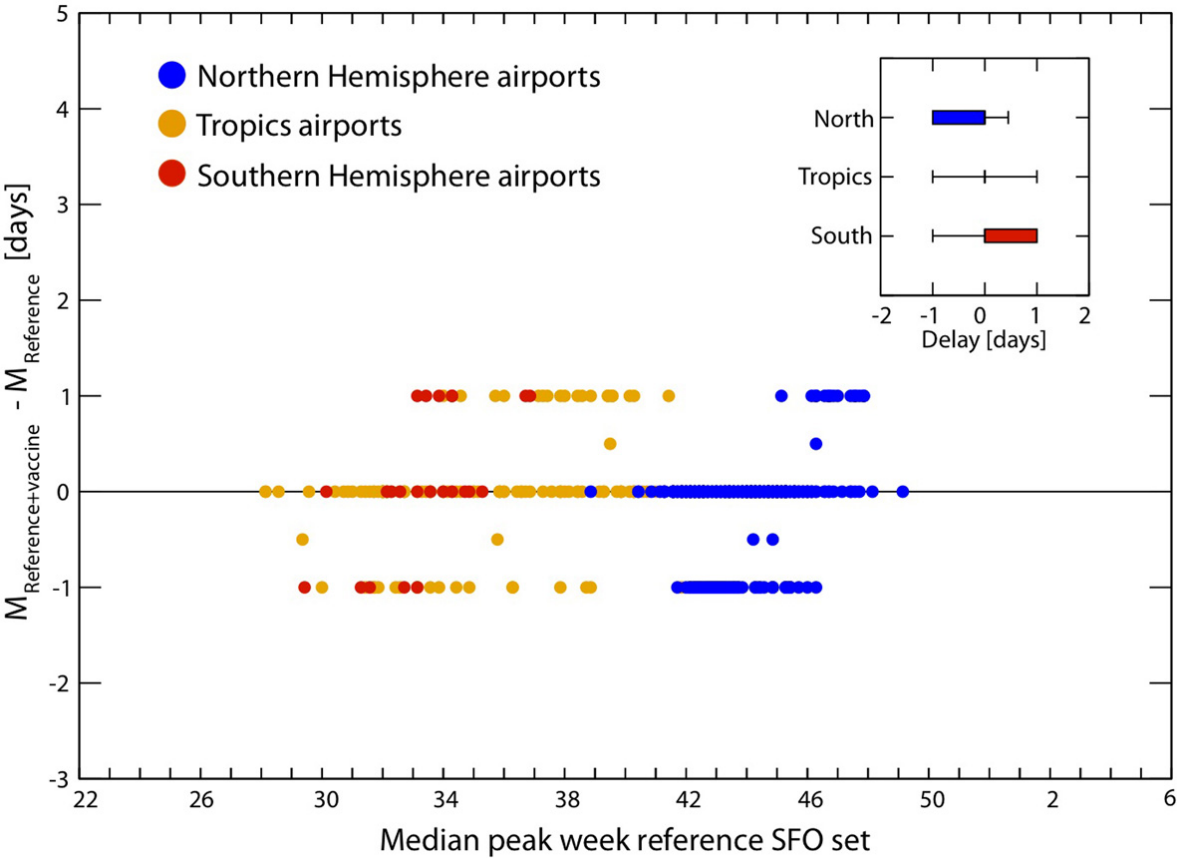
**Peak timing: effect of vaccination campaigns**. Difference in the median peak weeks in the reference stochastic forecast output (SFO) set with mass vaccination campaigns and the reference SFO set as a function of the median peak week in the reference SFO set, for the 500 busiest airports of the world. Dots are color-coded according to the corresponding airport's climatic zone. In the inset, we show the box plot indicating the distribution of the differences (in days) between the peak weeks of the two SFO sets. The differences were all limited to the minimal time scale used in the model (1 day) and thus were indistinguishable from stochastic fluctuations.

Although generally implemented too late to affect the timing of the pandemic in the northern hemisphere, the reactive vaccination campaigns implemented by several countries in that region might have helped to accelerate the decline of the pandemic and reduce its final attack rate. We considered the data available for 2010 on the start and coverage of the vaccination campaigns in those countries in which this measure was implemented, in order to calculate the daily distribution of vaccines and provide a more realistic description of the interventions adopted worldwide. The final vaccine uptake differed widely between countries in the northern hemisphere, ranging from 0.6% of the population in the Czech Republic to about half of the population or more in the northern European countries (Sweden, Finland, Iceland, Norway) and in Canada, thus resulting in a very heterogeneous picture (Table 2). Notwithstanding the large uptakes reached in some countries, the effect of the mass vaccination campaigns on the timing of the epidemic was negligible, as would be anticipated in the case of an early-peak scenario [68], because most of the vaccine doses were not deployed before November 2009. We integrated those data into the reference SFO dataset by generating a reference + vaccination dataset. We calculated the difference in the median value of the peak week between the reference + vaccination dataset and the reference SFO dataset for the 500 busiest transportation hubs worldwide and for a single seasonality value of *α*_min _= 0.65 (Figure 8). For all geographical locations, the difference in peak time between the reference + vaccination dataset and the reference SFO was no more than 1 day, with no significant changes in those countries with a larger fraction of immunized individuals.

The real data and the model output provided similar results, as expressed in terms of a negative correlation, which was not significant, between the peak week and the vaccine uptake (Table 4), indicating a larger uptake in those countries that experienced an earlier pandemic wave, such as Canada and the USA.

### Clinical attack rate and the effects of vaccination and pre-exposure immunity

The comparison of the absolute values of predicted attack rates with real data was hampered by the limited availability of accurate data on the total number of people infected by the 2009 H1N1 pandemic worldwide [99]. Surveillance data usually rely on the measure of the number of individuals with ILI symptoms who seek medical care, which leads to underestimation of the number of clinical cases because it does not account for those individuals with influenza who do not seek medical attention. By adjusting for consultation rates, current estimates of the epidemic size range from 1.8% for symptomatic cases in the UK [89], to 18% in France for the overall proportion of the infected population [100], to about 14% to 29% of the illness attack rate in the USA [101]. The large variation in these estimates is related to the intrinsic under-ascertainment of surveillance systems and to different healthcare-seeking behaviors, which may vary from country to country and may also change in time within the same population [89-91]. Additional estimates of the extent of the infection in a population were provided by serological analyses conducted during and immediately after the pandemic wave. Available studies measured overall attack rates of 19% in the UK during the first wave [78] and 36% after the second wave [102]; 21.5% in the USA, from data collected till early December 2009 [103]; and 11% in Hong Kong, from a survey running to the end of December 2009 [104]. However, these results are difficult to interpret given the sampling and timing biases of the serological analyses. However, the available evidence suggests that the incidence figures originally provided during and immediately after the outbreak dramatically underestimated the true number of overall infections [38,78,102].

In our model, the value of the attack rate depends on parameters that were prospectively unavailable for real-time forecast. The final epidemic size is non-trivially affected by the time and type of implementation of vaccination campaigns, by the level of pre-exposure immunity in the population, and by the proportions of asymptomatic infections, all information that became available only after the pandemic wave ended.

**Figure 9:**
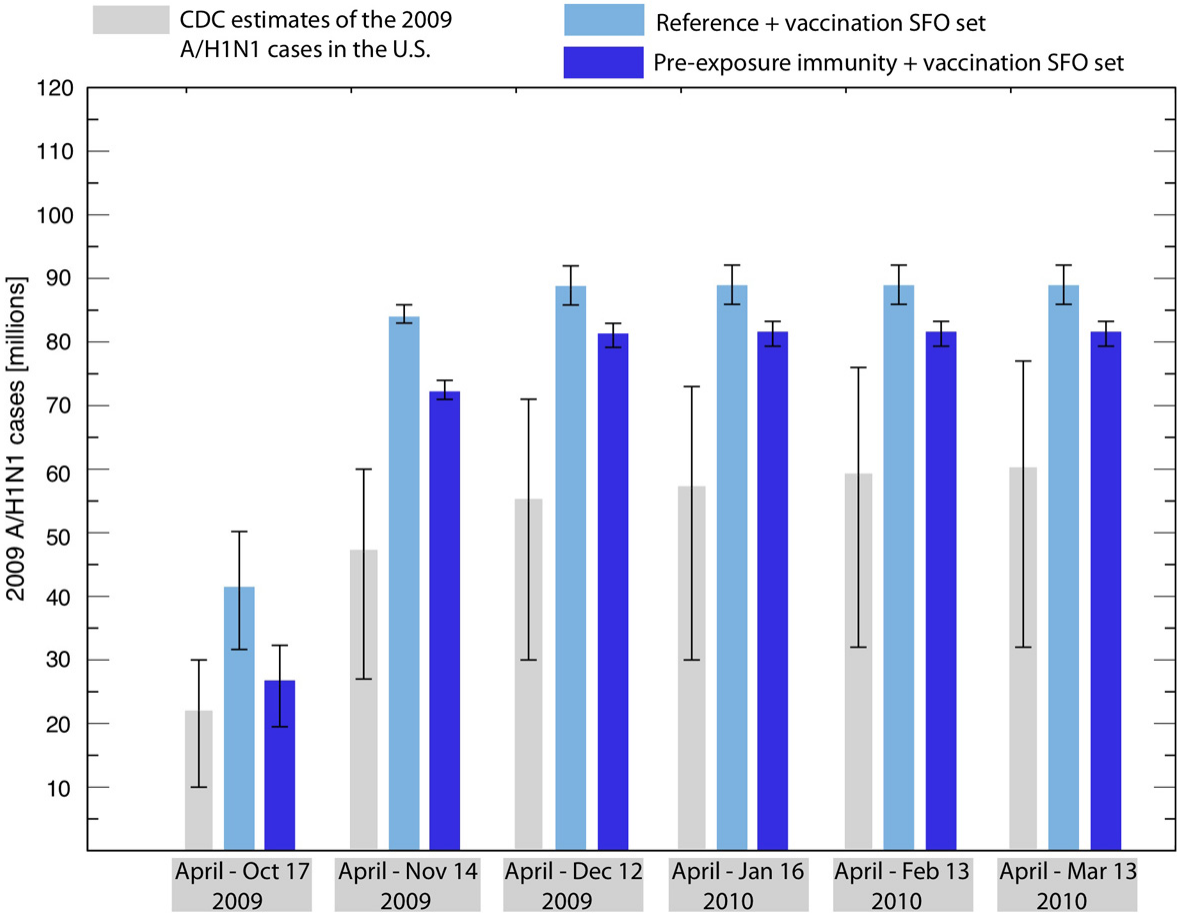
**Clinical attack rate in the USA**. The number of clinical A/H1N1 cases in the 2009 pandemic as estimated by the US Centers for Disease Control and Prevention (gray) and by two different stochastic forecast output (SFO) sets simulated by the Global Epidemic and Mobility (GLEAM) computational model, at six different dates between April 2009 and March 2010. The simulated results corresponded to the reference SFO set with vaccination and to the pre-exposure immunity SFO set with vaccinations, with the proportion of asymptomatic infections, *p_a_*, set to 45%. The bar indicates the median value and the error bar indicates the corresponding 95% reference range.

In an *a posteriori *analysis, we compared the figures estimated by the US Centers for Disease Control and Prevention (CDC) for the clinical attack rate in the USA [101] with the simulated results obtained by the reference SFO set and the pre-exposure immunity SFO set (Figure 9), both with reactive vaccination considered as intervention and the proportion of asymptomatic infections, *p_a_*, as 45%. For this, we followed the evidence provided by a few studies that showed how symptomatic infections in the H1N1 pandemic might have been less frequent than the rates known for seasonal influenza strains [62,63] (see Additional file 1, Figure S6, for results using *p_a _*= 33%).

**Figure 10:**
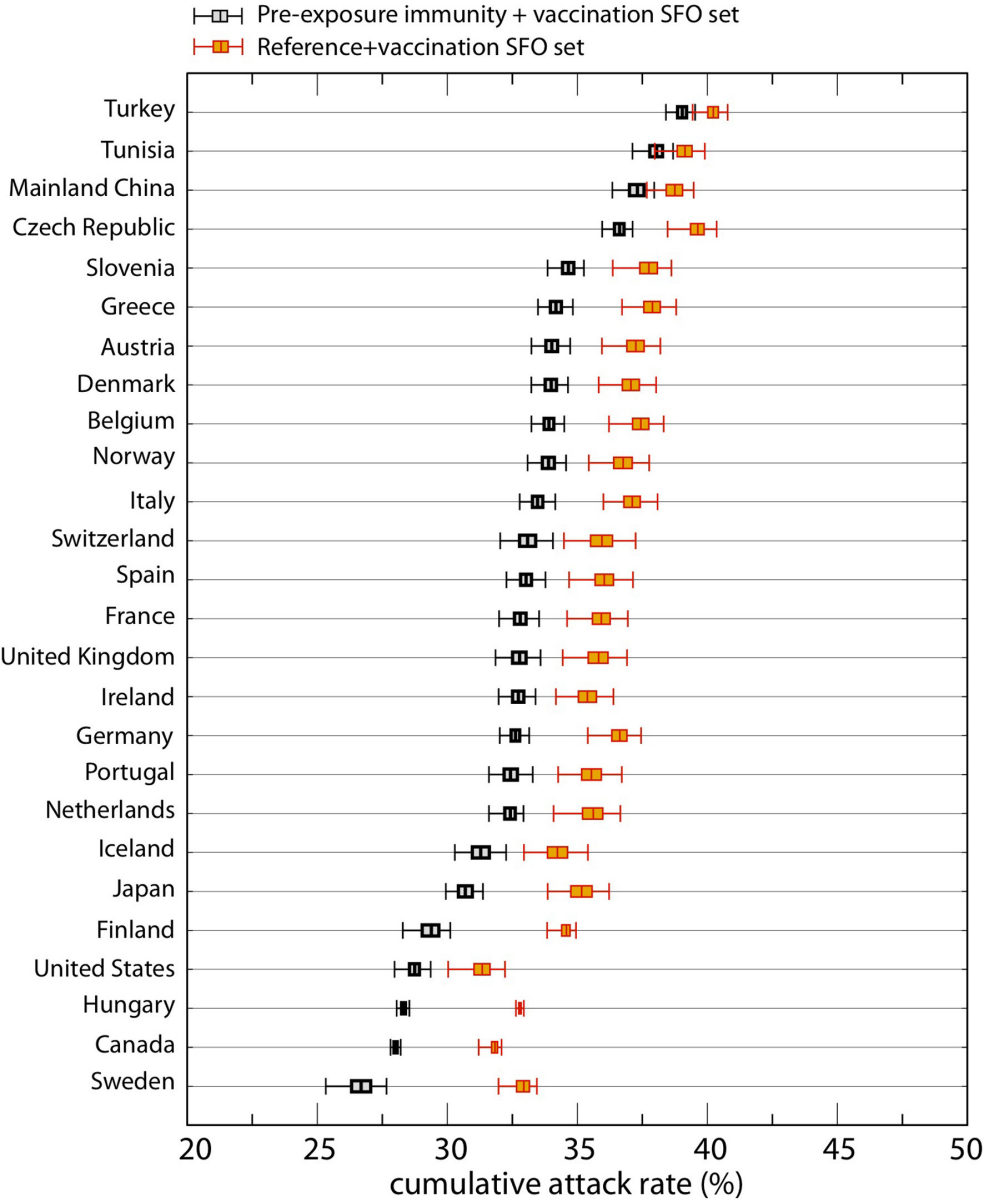
**Clinical attack rates in the northern hemisphere**. Estimated clinical attack rate of 26 selected countries in the northern hemisphere for the reference stochastic forecast output (SFO) set coupled with mass vaccination campaigns, and the pre-exposure immunity SFO set with vaccinations, both assuming a proportion of asymptomatic infections, *p_a_*, of 45%. The box plots indicate the 95% and 50% reference ranges, with the median value of the simulated attack rates obtained by the analysis of 2,000 stochastic realizations of the model for *α*_min _being 0.65.

We also calculated the estimated clinical attack rate for a selected number of countries in the northern hemisphere that implemented mass vaccination campaigns (Figure 10). Overall, the model SFO for the attack rates was higher than the values reported by serological analyses and several surveillance systems. However, they were close to the values estimated by surveillance systems in the USA and those provided by other modeled findings, which predicted 22%, 31%, and 48% of clinical attack rates for the USA [105], Italy [106], and France [100], respectively, in a scenario corresponding to our reference SFO set with vaccination. At the same time, sample sizes of several published serological studies were found to be too small to assess the validity of model predictions, except when large values of *R*_0 _were considered [107]. Therefore, it is difficult to make a direct comparison between the model results and currently available serological analyses. Furthermore, attack rates larger than those produced by agent-based models [105,106] have to be expected, because of the homogeneous assumption considered in the GLEAM model for the epidemic dynamics in each subpopulation. This issue has already been specifically addressed in a side-by-side comparison of GLEAM with an agent-based model simulating a pandemic-like event in Italy [42]. The results showed a systematic difference in the epidemic size of the two models, but without affecting the timing of the simulated epidemic.

**Figure 11:**
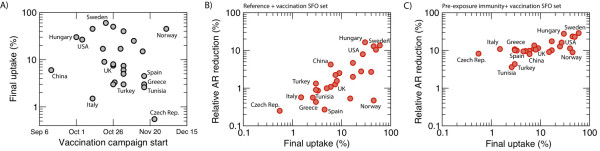
**Cumulative attack rate: effect of vaccination campaigns and pre-exposure immunity**. **(A) **Final vaccine uptake as a function of the mass vaccination starting date for the countries of the northern hemisphere (reported in Table 2). **(B) **Relative reduction of the final epidemic size in the reference stochastic forecast output (SFO) coupled with mass vaccination campaigns, with respect to the reference SFO, as a function of the final vaccine uptake. **(C) **Relative reduction of the final epidemic size in the pre-exposure immunity SFO coupled with vaccination campaigns, with respect to the reference SFO, as a function of the final uptake. The relative reduction of the epidemic size was calculated as the relative reduction of the maximum of the 95% reference range, obtained from 2,000 stochastic realizations, in the reference SFO set. In all SFO sets, we assumed the proportion of asymptomatic infections, *p_a_*, to be 33%.

While the reference + vaccination case allowed direct comparison of the model results with the available empirical data, the flexibility of the model also allowed us to assess the extent to which each component and/or intervention might affect the attack rate. The results of the previous subsection showed how massive vaccination campaigns had a very limited effect on the timing of the pandemic peak; however, the model predictions showed that their effect on the final attack rates was non-negligible [105], particularly in those countries where an early start was possible (Figure 11A, B). Overall, a significant positive correlation between vaccine uptake and a reduction in the final attack rate was evident in the model results (Table 4). As expected, the largest reductions in the set with vaccination were found for those countries that adopted prompt and rapid administration of vaccines (the USA, Hungary, and Sweden). In these cases, vaccination was able to achieve a relative reduction ranging from about 8% to 16%, if we consider the upper value of the reference range. A late start or a slow pace of vaccine distribution would be predicted to result in a much smaller reduction, as shown by the cases of Italy, the Czech Republic, and Norway, which were characterized by a low final uptake (Italy and Czech Republic) and/or late start to the vaccination campaign (Czech Republic and Norway). The average relative reduction obtained by considering all countries was 3.6%. However, it is important to note that estimates in reductions in the final attack rate from vaccinations are limited by the fact that the model does not incorporate age-specific transmission and vaccination strategies, therefore, the effect of vaccinations on a given age group could differ greatly from the average reduction in the final attack rate observed at the country level.

If we consider that a portion of the population had an initial degree of immunity, provided by cross-reacting antibodies elicited by previously circulating seasonal strains (see Methods section), the relative reduction in the final size of the epidemic increases considerably. We assessed the relative reduction obtained by comparing the pre-exposure immunity SFO set in which vaccination had also been implemented (that is, pre-exposure immunity + vaccination) with the reference SFO set (Figure 11C; also considered as a benchmark for the results shown in Figure 11B). A new MCML calibration that integrated data on pre-exposure immunity had to be performed to generate the corresponding set of SFO, because the initial conditions of the immunity profile of the population were changed with respect to the other SFO sets. Although this change did not affect the timing of the pandemic wave (Figure S5), the presence of pre-exposure immunity in older age groups reduced the effect of the disease in that population, in agreement with other modeling studies [100,106]. The values of the relative reduction increased by a factor of approximately 2 with respect to the reference + vaccination case for those countries experiencing the largest benefit from the massive vaccination campaigns (for example, USA, Hungary, and Sweden), and were considerably (about one order of magnitude) higher for the countries in which this benefit was extremely limited in the absence of pre-exposure immunity (for example, Czech Republic, Italy, and Norway). The relative variation is an overall result of the effects related to the start of the vaccination campaigns, peak timing, and the population distribution by age for each country owing to the pre-exposure immunity in older age groups. For instance, smaller reductions were seen in countries characterized by relatively young age profiles, such as Turkey and Tunisia.

### Antiviral treatment and pre-vaccination

During the 2009 A/H1N1 pandemic, only a few countries adopted the systematic use of antiviral drugs as a mitigation strategy: Canada, Germany, Hong Kong SAR, Japan, the UK, and the USA [82]. Furthermore, in those countries, the antiviral treatment was limited to the early stage of the outbreak, and the effort was not sustained in the later stages of the pandemic.

In the model, we considered the intervention applied to all countries having drug stockpiles available at the beginning of the outbreak [74], and for each country, we assumed this would occur until the country's stockpile was depleted. Antiviral treatment was considered in isolation on top of the reference SFO, and no vaccination was implemented. It should be noted that this is a 'what if' scenario informed by the data on antiviral stockpiles around the world.

**Figure 12:**
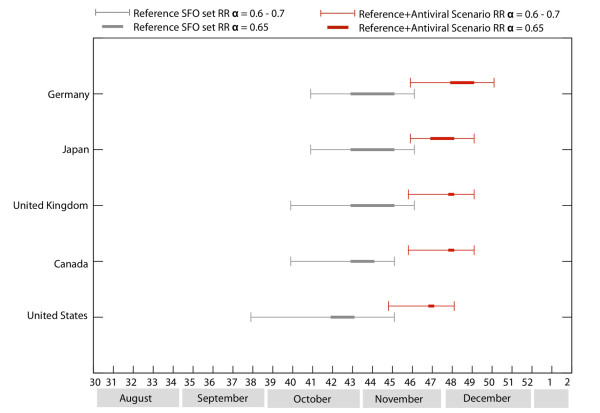
**Peak timing: effect of interventions by antiviral treatment**. Peak weeks of the epidemic activity in the reference stochastic forecast output (SFO) (gray) and in the antiviral scenario (red), for a set of countries in the northern hemisphere. The 95% reference ranges of the simulated peak week were obtained by analysis of 2,000 stochastic realizations of the model for three different values of the seasonal rescaling factor, *α*_min_: 0.6, 0.65, and 0.7.

We examined the hypothetical effect on the simulated activity peak times for a set of countries that adopted systematic antiviral treatments (Figure 12). Delays of about 3 to 4 weeks were obtained in all cases, assuming a systematic detection rate 30% for patients with influenza, with prompt administration of the drug. If compared with the start dates of the vaccination campaigns (Table 2), this slowing-down effect would have provided valuable time to immunize a larger fraction of the population before the pandemic wave reached its peak in those countries. A combination of antiviral treatment and mass vaccination campaigns would achieve much lower attack rates [68] at the end of the epidemic. However, assuming a lower and more realistic detection rate of clinical cases (5% or 10%), the delay in the pandemic peak would be only 1 to 2 weeks [68], allowing less time for implementing vaccination campaigns.

Moreover, the trade-off between the severity of the infection and the risk of inducing antiviral resistance has to be factored into the final decision considering the implementation of systematic treatment policies. In this hypothetical scenario, we considered the antiviral efficacy for transmission and reduction of the infectious period as that available in the modeling literature. As discussed in the Methods section, we acknowledge that there is an ongoing debate on the availability of the empirical evidence supporting these estimates [73].

We also evaluated the effectiveness of further hypothetical mitigation strategies by assuming vaccination had occurred before the epidemic onset [11,12,28] in different proportions of the population. Unlike the full immunity assumed in the pre-exposure immunity SFO ('all-or-nothing'), we considered here the same compartmentalization and assumptions as in the case of reactive vaccination, with relative efficacies against infection, and against transmission and development of clinical illness once infected (see Methods section). The GLEAM simulation results showed that a relative reduction of between 77% and more than 99% of the clinical attack rate would be achieved if 50% of the population were pre-vaccinated, thus successfully mitigating the epidemic spread. Smaller uptakes, of 20% and 30% of the population, would lead to relative reductions in the ranges of 20% to 28% and of 34% to 50%, respectively (Table 5).

**Table 5:** Effects of pre-vaccination.

Country	Relative reduction in epidemic size, %^a^		
	
	Pre-vaccination 20%	Pre-vaccination 30%	Pre-vaccination 50%
China	27.3 to 28.2	46.3 to 48.2	98.9 to 99.3
Hungary	23.2 to 24.4	38.6 to 39.2	87.9 to 90.0
United States	20.5 to 21.8	35.2 to 36.2	92.4 to 95.5
Canada	21.5 to 22.6	37.5 to 38.3	93.4 to 95.6
Italy	23.0 to 24.4	38.3 to 39.0	90.2 to 91.8
Japan	23.4 to 26.8	38.1 to 40.1	85.5 to 89.8
Israel	22.9 to 24.8	37.8 to 38.8	84.3 to 86.4
France	22.0 to 24.2	36.6 to 38.1	84.4 to 86.8
Sweden	23.1 to 24.8	38.2 to 39.0	86.3 to 88.6
UK	22.2 to 24.6	36.6 to 38.4	84.7 to 88.4
Germany	22.6 to 24.2	37.7 to 38.6	88.9 to 90.6
Portugal	22.7 to 24.5	37.1 to 38.6	88.3 to 92.4
Finland	22.9 to 24.7	37.7 to 38.7	84.5 to 87.3
Austria	21.6 to 23.5	35.8 to 37.1	81.7 to 84.1
Ireland	22.0 to 24.0	36.2 to 37.6	83.0 to 86.6
Denmark	21.5 to 23.5	35.6 to 37.1	81.7 to 85.0
Turkey	26.5 to 27.0	46.4 to 49.6	95.4 to 95.8
Iceland	20.6 to 24.8	33.9 to 38.6	78.8 to 87.8
Belgium	23.2 to 24.8	38.6 to 39.4	89.1 to 91.1
Slovenia	21.5 to 23.3	35.3 to 36.8	76.4 to 81.1
Netherlands	21.7 to 24.7	36.3 to 38.4	85.5 to 89.5
Switzerland	21.0 to 24.0	34.4 to 36.8	77.5 to 81.9
Spain	22.4 to 23.7	37.7 to 39.0	91.7 to 93.7
Greece	23.2 to 24.8	38.6 to 39.5	86.1 to 87.7
Tunisia	24.5 to 25.7	41.0 to 42.1	90.9 to 92.0
Czech Republic	24.8 to 25.6	42.0 to 44.9	89.7 to 90.6
Norway	23.1 to 24.7	37.9 to 38.9	85.9 to 88.7

^a^Relative reduction of the epidemic size, using different hypothetical pre-vaccination scenarios, in the countries of the northern hemisphere in which vaccines were available during the 2009 to 2010 winter season. The relative reduction in the epidemic size was calculated as the relative reduction of the maximum of the 95% reference range, obtained from 2,000 stochastic realizations, in the reference stochastic forecast output set.

### Sensitivity analysis on the epidemiological parameters

In this analysis, we explored the effects induced by changes in the values of the epidemiological parameters considered in the model, including assumptions and estimated values. First, we focused on the sinusoidal rescaling of the reproductive number to account for seasonal effects. We assumed a different setting in which the maximum value of the rescaling function, *α*_max_, was set to 1 [4,9] instead of 1.1 [2,108] thus, the maximum value reached by *α*(*t*)*R*_0 _in the temperate regions during wintertime is equal to the reference value of the reproductive number in the Tropics, *R*_0_.

**Figure 13:**
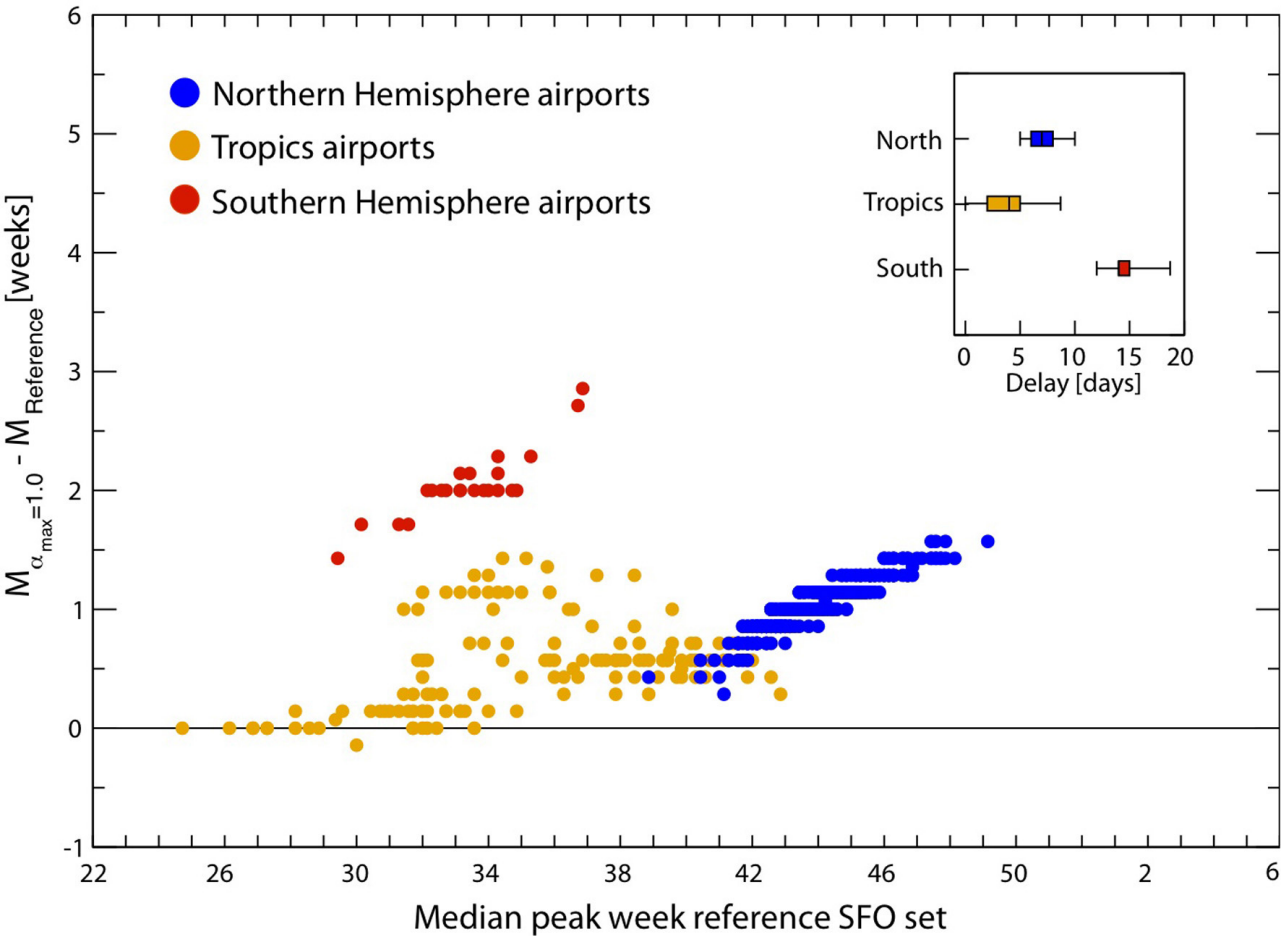
**Peak timing: effect of changes in the maximum seasonal rescaling**. Difference in the median peak weeks in the reference stochastic forecast output (SFO) set, with *α*_max _= 1.1 and *α*_max _= 1.0, for the 500 busiest airports, as a function of the median peak week in the reference SFO set. Dots are color-coded according to the corresponding airport's climate zone. In the inset, we show the box plot indicating the distribution of the differences (in days) between the peak week of the reference SFO set and the SFO set with *α*_max _= 1.0. Differences were fairly limited and generally fell within a period of 2 weeks.

We computed the shift induced in the median peak week by considering the *α*_max _= 1 scenario, compared with the reference SFO set where *α*_max _= 1.1, for the 500 busiest airports in the database (Figure 13; the color codes indicate the climate region of each airport). The change in the value produced a delay in the activity peak of every country that was never greater than 3 weeks, with 98.6% of the airports experiencing a delay of 2 weeks at most, and only countries in the southern hemisphere witnessing a delay exceeding 2 weeks. Given that delays of less than 2 weeks are within the reference range of our simulation results for the reference SFO set, this change would have a limited effect on the validity of our predictions for the timing of the pandemic wave in the northern hemisphere (Figure 5).

Second, we investigated the role of our estimate of the seasonal effects from the initial pandemic international invasion. Instead of estimating the value of *α*_min _in the range of 0.6 to 0.7 from the correlation procedure described in the calibration subsection, we assumed *α*_min _= 0.1, as in the case of seasonal influenza [9]. In this case, the simulations predicted an epidemic peak at the beginning of 2010 for almost all countries in the northern hemisphere, thus resulting in a delay of about 1.5 months with respect to the initial predictions of the baseline case (Figure S7). These results highlight the importance of obtaining a correct estimate of the strength of seasonal effects in order to provide accurate predictions.

Finally, we also investigated the effect of changes in the assumed parameters concerning asymptomatic infections, that is, the relative fraction of asymptomatic cases, *p_a _*and their relative transmissibility, *r_β_*. A broad exploration of parameters and a thorough sensitivity analysis has been provided in a previous study [2]. For the sake of completeness, we provide an enlarged sensitivity analysis of the parameter *r_β _*(see Additional file 1K), which is generally assumed in modeling studies to be equal to 50% [23,24,27]. We found that the predictions obtained in the baseline SFO set for the timing of the pandemic influenza peak and the illness attack rate were very robust against changes of *r_β_*, even in the extreme case of *r_β _*= 10% (Figure S8).

### Sensitivity analysis of data knowledge and integration

The sensitivity analysis of most epidemic models focuses only on the parameters describing the disease. However, in a large-scale computational model, the integration and assimilation of data on census, mobility, and other demographic factors has to deal with issues related to the quality and completeness of the data. The sensitivity analysis of the model results with regard to the incompleteness or poor quality of those 'structural' data is thus extremely important. We tested this aspect by assessing whether the full complexity of the real mobility data considered in GLEAM would be essential to obtain the SFO presented in the previous subsection, or if a simplified version of the model would allow similar results.

Other approaches have considered only one transportation mode (air travel) and included a limited number of airports, ranging from 52 to 500 [4,6,9,14,29,43]. A recent study compared the spread of influenza at the global level by considering different samples obtained from the full OAG (Official Airline Guide) [52] database of 2000 [41]. Its results have shown that samples of the 200 to 300 largest airports in the world would reproduce fairly well the backbone of spreading at the global and regional scales. Although we agree with previous studies which state that considering partial datasets is informative for the overall theoretical analysis of general spreading features, we tested the performance of partial datasets in providing reliable SFO sets at the country or city scale.

We performed the simulations of the 2009 A/H1N1 pandemic on a version of GLEAM that integrated a partial dataset restricted to the top 500 worldwide transportation hubs, ranked by their traffic, with no short-range mobility considered. This greatly reduced the geographic extension of the model, and unfortunately, most of the top 500 airports lie in Europe and North America, with very few belonging to African countries. Of 220 countries, only 126 were within the reach of the model, thus corresponding to a 43% drop from the full GLEAM model. We report a breakdown by continent of the countries that can still be analyzed using only 500 airports (Table 6). The simulations considered the reference SFO set both in the mobility-sampled and the original versions of the GLEAM model.

**Table 6:** Geographic resolution of GLEAM with the full database and with the top 500 airports.

Continent	Countries in the full database, n	Countries in the top 500 database, n	Relative reduction, %
Asia	44	36	18
Europe	45	31	31
Americas	49	32	35
Africa	56	20	64
Oceania	26	7	73

Because this analysis modified the structure of the model, we performed a new calibration, using a new estimate of the reproductive number (*R*_0_) of 1.5, and a value of *α*_min _in the range 0.8 to 0.9. The calibration procedure was still based on the same dataset of arrival times used in the reference SFO set, because the corresponding countries were covered by the sampled mobility network.

**Figure 14:**
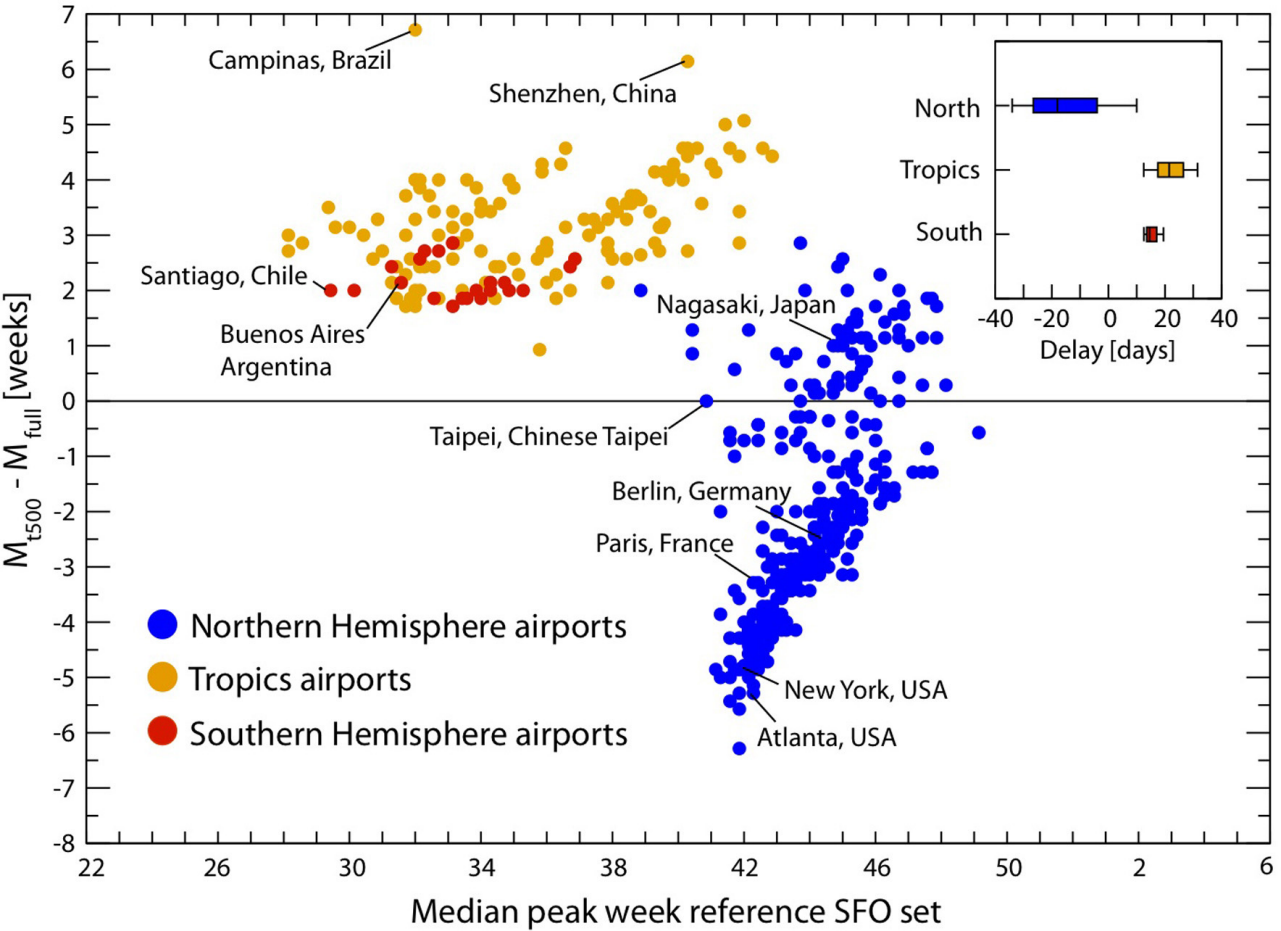
**Peak timing: effect of sampling of the mobility network limited to the top 500 airports**. Difference in the median peak weeks in the reference stochastic forecast output (SFO) set, where the full mobility dataset was considered, and the top 500 scenario, for the 500 busiest airports, as a function of the median peak week in the reference SFO set. Dots are color-coded according to the corresponding airport's climate zone. In the inset, we show the box plot indicating the distribution of the differences (in days) between the peak week of the reference SFO set and the SFO set considering only the top 500 airports. The differences were considerable, with median differences of about 3 weeks.

As a consequence of the low transmissibility and the small seasonal effect, the spread of the pandemic on the sampled network appeared to be faster in the northern hemisphere, leading to an earlier activity peak for most of the airports, except for a few cases, which showed a delay of up to 4 weeks compared with the full database scenario, because of their reduced connectivity in the sampled network (Figure 14). The largest airports in Europe and North America, such as Paris in France and Atlanta in the USA, experienced the largest shift in the activity peak, ranging from 20 to 45 days earlier than the corresponding timing of the SFO that integrated the full dataset. In the southern hemisphere and the Tropics, the activity peak was postponed with respect to the reference SFO set. For large airports, the delay was limited to 2 weeks, as shown in the cases of Santiago in Chile and Buenos Aires in Argentina; however, for some less connected airports, such as Shenzhen in China and Campinas in Brazil, the delay in the influenza activity peak was 45 days.

Although the top 500 airports gather about 80% of the worldwide air traffic, the differences in the median peak times are clearly non-negligible (Figure 14). A specific application to a real-world epidemic is thus able to show how the global backbone of invasion can be strongly affected by the partial sampling of the mobility network, owing to the interplay of different parameters, such as the presence of loops, local connectivity, seasonal effects, and the real and effective (that is, measured on the sampled network) distance of the location from the seed of the outbreak. In addition, a limited version of the model may not be applicable to a specific real epidemic, given its partial coverage of the locations and countries in the world, as would be the case where the initial seed of the outbreak belongs to a region not included in the data integrated into the model. Indeed, in the top 500 airports, the sampling of the mobility network restricts the choice of the seed location to Mexico City, which is the major airport found close to the original outbreak location. Therefore, the results (Figure 14) are affected by the following two effects, which occur because of the restriction of the analysis to the top 500 airports: the change in the mobility network structure and the change in the seed location, as forced by the lack of resolution in the sampled mobility network. For this reason, we analyzed the change in peak time as a function of the initial condition in Mexico City in the case of the full mobility network (see Additional file 1L). The results showed how the reduction of the full mobility dataset has considerable consequences on the timing of the pandemic in the various locations discounted of the effects induced by the initial condition resolution. Overall, the peak time shift between the full database scenario and the sampled database scenario, which both had the seed in Mexico City, ranged from 69 days earlier up to 20 days later (Figure S9).

**Figure 15:**
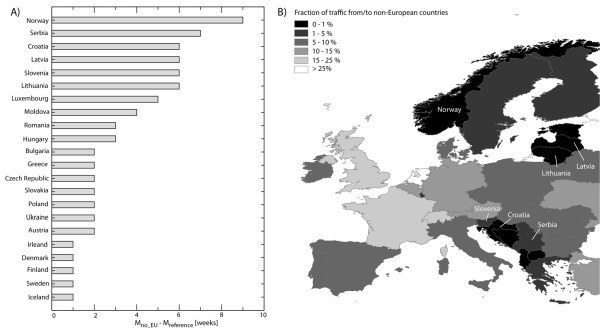
**Peak timing: effect of intra-EU mobility connections**. **(A) **Delay of the A/H1N1 pandemic peak time in the scenario with no intra-EU air connections, with respect to the reference stochastic forecast output (SFO) set, for 22 European countries. **(B) **Map of Europe, showing for each country the fraction of traffic directed to and from non-European countries. Countries with small extra-European connections experienced the largest delay in the A/H1N1 pandemic peak time. The map was made exclusively for this manuscript and is not subject to copyright.

To examine the effects of further model simplifications, we also explored the results of our simulations after the removal of mobility connections between the European countries, keeping the structure of the model untouched in the rest of the world. This led to a systematic delay in the peak of activity in all European countries compared with the full model, because the removal of intra-European Union (EU) connections meant that the only possible source of infection could be from outside the EU. The delay in median values of the peak time ranged from 1 week to 9 weeks (Figure 15A), and was strongly correlated with the travel flows of passengers flying into a given country from outside Europe (Figure 15B). Countries with a small extra-EU traffic fraction, such as Norway, Slovakia, Croatia, and Slovenia, experienced the largest delay. On the other side of the spectrum, some countries, such as the UK and France (not shown) experienced negligible or no changes, because their primary source of infection was from North America.

## Conclusions

In this study, we examined the application of GLEAM, a global stochastic simulation model of epidemic spread based on real data of human population distribution and mobility, to the 2009 A/H1N1 pandemic. We analyzed, in real time, the pandemic emergency that led to the publication in summer 2009 of the predicted timing for the pandemic wave in the countries in the northern hemisphere for the fall/winter period. Using surveillance data from various monitoring and virologic sources, we have provided a validation of the SFO of the GLEAM model for the unfolding of the A/H1N1 pandemic in 2009. Our findings indicate very good agreement in the predicted timing for a large variety of countries, including those with underdeveloped surveillance schemes, and for intra-country spatial scales. The results are encouraging in advocating the use of large-scale computational approaches in providing real-time forecast and scenarios of epidemic outbreaks. If the appropriate MCML calibration is performed, the SFOs are very stable against changes in epidemiological parameters that are difficult to estimate for an emerging virus, such as the asymptomatic proportion of the population and its relative infectiousness. Changes in those parameters are generally absorbed by the rescaling of the key disease parameters in a self-consistent way. However, the model output shows strong dependence on the accuracy of the initial conditions and the mobility network considered. This highlights the need for a detailed level of description of human mobility and population distribution in the world in order to achieve reliable predictions at a high-resolution scale. We also considered additional scenarios to allow more realistic simulation of the pandemic event worldwide, based on detailed data of country-based interventions and population initial immunity profiles, which became available throughout and after the outbreak. Consequently, accurate data should be rapidly available during the initial phase of the outbreak in order to allow careful calibration of the model, and close collaboration with public-health officials should allow careful consideration of possible intervention scenarios to support policy decisions for contingency planning at both country and global levels.

## Abbreviations

ARI: Acute respiratory infection; GLEAM: Global Epidemic and Mobility; ILI: Influenza-like illness; MCML: Monte Carlo Maximum Likelihood; MCM: Mathematical and Computational Model; SFO: Stochastic Forecast Output.

## Competing interests

The authors declare that they have no competing interests.

## Authors' contributions

All authors participated in the numerical analysis and development of the model. MT, VC and AV designed the study, analyzed data, and wrote the manuscript. All authors reviewed and approved the final version of the manuscript.

## Pre-publication history

The pre-publication history for this paper can be accessed here:

http://www.biomedcentral.com/1741-7015/10/165/prepub

## Supplementary Material

Additional file 1 **Real-time numerical forecasts of global epidemic spreading: case study of 2009 A/H1N1pdm. Supporting Information**. List of data sources and sensitivity analyses on model's structure and epidemic parameters. Click here for file
