# Supplementary material for: Real-time numerical forecast of global epidemic spreading: case study of 2009 A/H1N1pdm
Source: BMC Med. 2012 Dec 13;10:165. doi: 10.1186/1741-7015-10-165 (PMC3585792; doi:10.1186/1741-7015-10-165)
Supplement: Additional file 1 — Real-time numerical forecasts of global epidemic spreading: case study of 2009 A/H1N1pdm. Supporting Information. List of data sources and sensitivity analyses on model's structure and epidemic parameters. [file 1741-7015-10-165-S1.PDF]

Additional File 1

Real time numerical forecast of global epidemic spreading: case study of 2009 A/H1N1pdm – Supporting Information

Authors

M. Tizzoni[1,£], P. Bajardi[1,2], C. Poletto[1], J.J. Ramasco[3], D. Balcan[1], B. Gonçalves[4], N. Perra[4], V. Colizza[5,6,7], A. Vespignani[4,7,8]

[1] Computational Epidemiology Laboratory, Institute for Scientific Interchange (ISI), Torino, Italy
[2] Centre de Physique Théorique (CNRS UMR 6207), Marseille, France
[3] Instituto de Física Interdisciplinar y Sistemas Complejos IFISC (CSIC-UIB), Palma de Mallorca, Spain
[4] Department of Health Sciences and College of Computer and Information Sciences, Northeastern University, Boston MA 02115 USA
[5] INSERM, U707, Paris, France
[6] UPMC Université Paris 06, Faculté de Médecine Pierre et Marie Curie, UMR S 707, Paris, France
[7] Institute for Scientific Interchange (ISI), Torino, Italy
[8] Institute for Quantitative Social Sciences at Harvard University, Cambridge MA, 02138 USA

[£] Current address: Mathematical Modeling Services, UBC Centre for Disease Control, 655 West 12th Ave., Vancouver BC V5Z 4R4, Canada.

A. Commuting networks data

Our commuting databases have been collected from the Offices of Statistics of 30 countries in the 5 populated continents. The full dataset summary is reported in Table S1 and includes more than 78,000 administrative regions and over five million commuting flow connections among them (see [1]). The definition of administrative unit and the granularity level at which the commuting data are provided enormously vary from country to country. Even within a single country the actual extension, shape, and population of the administrative divisions are usually a consequence of historical reasons and can be strongly heterogeneous.

Such heterogeneity renders the efforts to define a universal law describing commuting flows likely to fail. In order to overcome this problem, and in particular to define a data-driven short range commuting for GLEaM, we used the geographical census areas obtained from a Voronoi tessellation as the elementary units to define the centers of gravity for the process of commuting. We have therefore mapped the different levels of commuting data into the geographical census areas formed by the Voronoi-like tessellation procedure around the main transportation hubs. The mapped commuting flows can be seen as a second transport network connecting subpopulations that are geographically close. This second network can be overlaid on the Worldwide Airport Network in a multi-scale fashion to simulate realistic scenarios for disease spreading.

Being the census areas relatively homogeneous and self-similar allows us to estimate a gravity law that successfully reproduces the commuting data obtained across different continents, and provides

us with estimations for the possible commuting levels in the countries for which such data are not available as in Ref. [1].

Table S1 – Commuting networks in each country.

Continent	Country	Administrative units	Connections
Europe	Austria	99	1,886
	Belgium	589	71,528
	Denmark	248	20,990
	Finland	348	22,484
	France	36,602	1,984,825
	Germany	439	46,465
	Greece	1,034	26,525
	Hungary	3,140	45,403
	Italy	8,101	446,056
	Netherlands	504	15,120
	Norway	430	29,285
	Portugal	308	27,694
	Slovenia	192	3,690
	Spain	52	826
	Sweden	290	31,438
	Switzerland	2,896	185,172
	UK	10,608	1,531,263
North America	Canada	3,845	19,202
	Mexico	2,443	63,678
	USA	3,141	163,053
Central America	El Salvador	262	11,438
	Nicaragua	153	4,786
South America	Chile	342	29,410
	Colombia	1,101	18,044
Asia	Hong Kong	18	306
	Japan	2,364	302,339
	Korea	136	2,567
	Taiwan	350	21,170
Oceania	Australia	674	27,688

New Zealand	72	2,991

B. Data on the A/H1N1 worldwide spreading

In order to estimate the reference value of the reproductive number in the Tropics region, we used a Monte Carlo likelihood analysis based on the arrival times of the first infected case in the countries seeded by the pandemic source, that is, Mexico. This is the only information that we used as input to calibrate the model.

We focused on 12 countries seeded by Mexico for which we could find a clear description of the first confirmed A/H1N1pdm case. We consulted official data and reports, when available. When the information from official sources was incomplete, we relied on news from the local press.

Table S2 reports the arrival dates of the first confirmed case in the 12 countries, defined as the date of onset of symptoms or the date of reporting, if the onset of symptoms was not available.

Table S2 – Chronology of arrival dates in 12 countries seeded by Mexico.

Country	Arrival date
**United States**	March 28, 2009
**Canada**	April 11, 2009
**Colombia**	April 14, 2009
**El Salvador**	April 19, 2009
**Germany**	April 20, 2009
**France**	April 23, 2009
**United Kingdom**	April 24, 2009
**Spain**	April 25, 2009
**Cuba**	April 25, 2009
**Costa Rica**	April 25, 2009
**Netherlands**	April 27, 2009
**Guatemala**	May 1, 2009

C. Natural history of influenza with pharmaceutical interventions

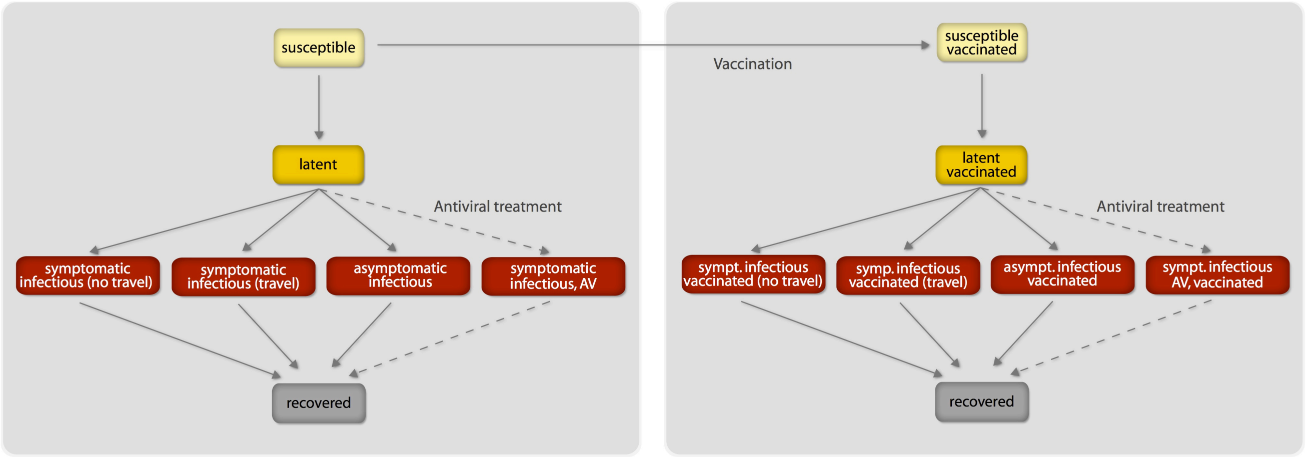

Figure S1. Compartmental structure in each subpopulation with pharmaceutical interventions. Antiviral treatment is assumed to be administered to a fraction p_{AV} of the symptomatic infectious individuals within 1 day from the onset of symptoms, according to the drugs availability in the country. It reduces the infectiousness by the antiviral efficacy AVE_I and shortens the infectious period of 1 day. If vaccines are available, a fraction of the susceptible population enters the susceptible vaccinated compartment each day. A similar progression to the baseline compartmentalization is considered if infection occurs. However, the vaccine reduces the susceptibility of the vaccinated susceptible with an efficacy VE_S, the probability of developing symptoms if infection occurs with an efficacy VE_D, and their transmission rate while infectious with an efficacy VE_I. All transition process are modeled through multinomial processes.

D. Time dependence of the reproduction number in the baseline SFO set

The model reproduces seasonality by means of a sinusoidal rescaling of R_0, resulting in an effective time dependent reproductive number R(t) in the Northern and Southern Hemisphere. Figure S2 shows the time dependence of the seasonally rescaled reproduction number for the estimated values of R_0 and α_{min} in the A/H1N1 pandemic baseline SFO set.

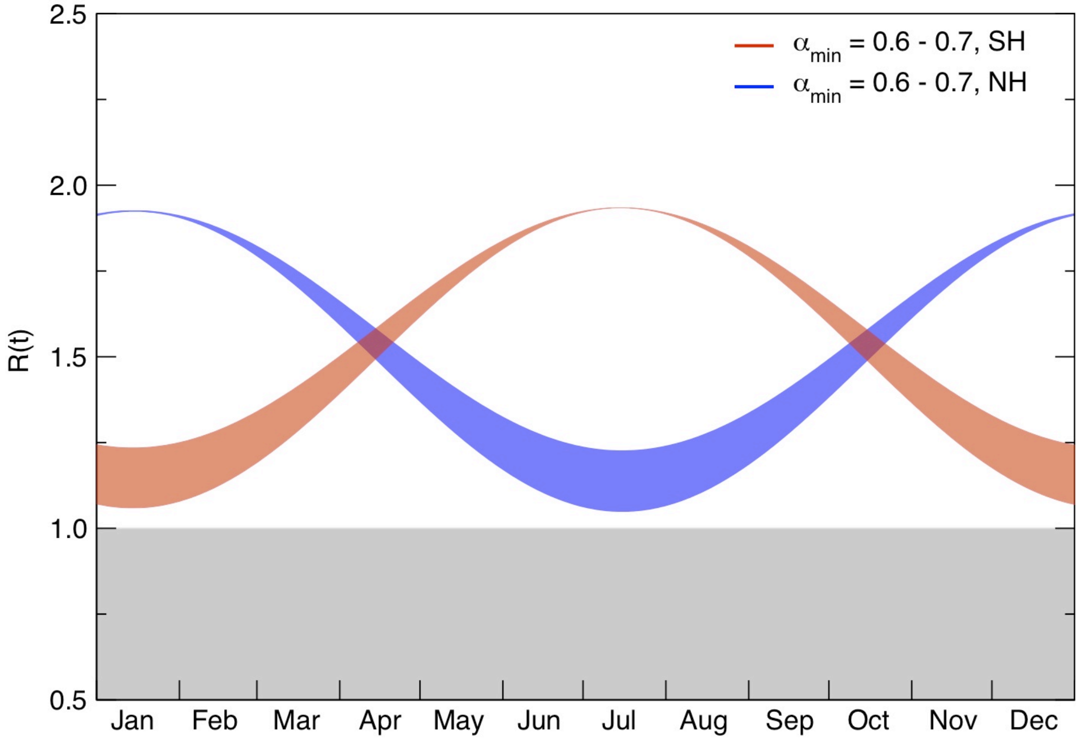

Figure S2. Seasonally rescaled reproductive number for R_0 = 1.75. Time dependence of the seasonally rescaled reproduction number in the Northern (blue shaded area) and Southern (red shaded area) Hemispheres, for the estimated values of R_0 = 1.75, and α_{min} = 0.6 and 0.7. The grey area highlights the region with $R<1$, where a sustained transmission can not occur.

E. Surveillance and vaccination data sources

Table S3 - Influenza surveillance data sources.

We collected data on influenza incidence during the 2009 – 2010 winter wave from the surveillance monitoring systems of 48 countries worldwide. Here we list the data sources of all countries, with their Internet address, when applicable. Our search strategy was mainly based on the availability and accessibility of surveillance data through internet websites. Our main goal was to collect the largest possible sample of surveillance data from North America, Europe, and Asia, browsing the websites of national agencies that provided surveillance data in a clear format, with a comprehensive description of the adopted surveillance methods.

North America

Country	Data type	Source	Internet address
Mexico	H1N1 laboratory confirmed cases	Secretaria de Salud, Mexico	http://portal.salud.gob.mx/
Canada	% ILI visits	Public Health Agency of Canada	http://origin.phac-aspc.gc.ca/fluwatch/
United States	% ILI visits	Centers for Disease Control and Prevention	http://cdc.gov/flu/weekly

Europe

Country	Data type	Source	Internet address
Albania	ARI incidence	Euro Flu	http://www.euroflu.org
Austria	ARI incidence	ECDC	http://ecdc.europa.eu
Belgium	ILI incidence	ECDC	http://ecdc.europa.eu
Bulgaria	ARI incidence	ECDC	http://ecdc.europa.eu
Croatia	ILI incidence	Euro Flu	http://www.euroflu.org
Czech Republic	ILI incidence	ECDC	http://ecdc.europa.eu
Denmark	ILI incidence	ECDC	http://ecdc.europa.eu
Estonia	ILI incidence	ECDC	http://ecdc.europa.eu
Finland	H1N1 laboratory confirmed cases	National Institute of Health	http://www.thl.fi

France	ILI incidence	Reséau Sentinelles	http://www.sentiweb.org/
Germany	H1N1 laboratory confirmed cases	Koch Institut	http://influenza.rki.de
Greece	ILI incidence	ECDC	http://ecdc.europa.eu
Hungary	ILI incidence	ECDC	http://ecdc.europa.eu
Iceland	ILI incidence	ECDC	http://ecdc.europa.eu
Ireland	ILI incidence	ECDC	http://ecdc.europa.eu
Italy	ILI incidence	Istituto Superiore di Sanità	http://www.iss.it/iflu/
Lithuania	ILI incidence	ECDC	http://ecdc.europa.eu
Latvia	ILI incidence	ECDC	http://ecdc.europa.eu
Moldova	ILI incidence	ECDC	http://ecdc.europa.eu
Netherlands	ILI incidence	ECDC	http://ecdc.europa.eu
Norway	ILI incidence	ECDC	http://ecdc.europa.eu
Poland	ILI incidence	ECDC	http://ecdc.europa.eu
Portugal	ILI incidence	ECDC	http://ecdc.europa.eu
Romania	ILI incidence	Euro Flu	http://www.euroflu.org
Serbia	ILI incidence	Euro Flu	http://www.euroflu.org
Slovakia	ILI incidence	ECDC	http://ecdc.europa.eu
Slovenia	ILI incidence	ECDC	http://ecdc.europa.eu
Spain	ILI incidence	Sistema de Vigiliancia de la Gripe en Espana	http://vgripe.isciii.es/gripe/inicio.do
Sweden	ILI incidence	ECDC	http://ecdc.europa.eu
Switzerland	ILI incidence	Euro Flu	http://www.euroflu.org
Turkey	ILI incidence	Euro Flu	http://www.euroflu.org
United Kingdom	ILI incidence	Health Protection Agency	http://www.hpa.gov.uk
Ukraine	ARI incidence	Euro Flu	http://www.euroflu.org

**Asia**			
**Country**	**Data type**	**Source**	**Internet address**
Georgia	ILI incidence	Euro Flu	http://www.euroflu.org

India	H1N1 laboratory confirmed cases	Ministry of Health	http://www.mohfw-h1n1.nic.in
Indian regions	H1N1 laboratory confirmed cases	WHO South East Asia Regional Office	http://www.searo.who.int/
Japan	# ILI patients per sentinel	Infectious Disease Surveillance Center	http://idsc.nih.go.jp/
Kyrgyzstan	ARI incidence	Euro Flu	http://www.euroflu.org
Korea (Republic of)	% ILI visits	Center for Disease Control	http://cdc.go.kr/
Mainland China	% ILI visits	Ministry of Health	
Mongolia	ILI incidence	National Influenza Center	http://www.flu.mn/eng/
Russian Federation	ARI incidence	Euro Flu	http://www.euroflu.org
Uzbekistan	ILI incidence	Euro Flu	http://www.euroflu.org

**North Africa and Middle East**			
**Country**	**Data type**	**Source**	**Internet address**
Israel	ILI incidence	Euro Flu	http://www.euroflu.org
Morocco	ILI incidence	Ministry of Health	http://srvweb.sante.gov.ma
Oman	# ILI cases	Ministry of Health	http://www.moh.gov.om/a_h1n1

Table S4 - Vaccination data.

We collected data on the vaccination campaigns against the H1N1 pandemic conducted in 27 countries of the Northern Hemisphere. Here, we list the full list of our data sources that comprise academic and institutional references, with their Internet address when applicable.

Country	Source	Internet address
China	Chinese Ministry of Health, as reported by Xinhua News Agency	http://news.xinhuanet.com/english2010/health/2010-03/02/c_13194419.htm
Hungary	National Center for Epidemiolgy	http://www.oek.hu/oek.web
United States	US Center for Disease Control and Prevention	http://www.cdc.gov/mmwr/preview/mmwrhtml/mm5912a2.htm
Canada	Public Health Agency of Canada	http://www.phac-aspc.gc.ca/
Italy	Italian Ministry of Health	http://www.nuovainfluenza.salute.gov.it/
Japan	Japanese Ministry of Health, Labour and Welfare	http://www.mhlw.go.jp/shingi/2010/03/dl/s0312-12a.pdf
Israel	Israeli Ministry of Health, as reported by the Jerusalem Post	http://www.jpost.com/HealthAndSci-Tech/Health/Article.aspx?id=168232
France	French Ministry of Health	http://www.pandemie-grippale.gouv.fr/contexte/les-dernieres-actualites/grippe-a-h1n1-point-presse-sur-le-bilan-de-la-vaccination.html
Sweden	Swedish Institute for Communicable Disease Control	http://www.smi.se/publikationer/smis-nyhetsbrev/influensarappor

		ter/sasongen-20092010/
United Kingdom	UK Department of Health	http://www.dh.gov.uk/en/Publicationsandstatistics/Publications/PublicationsStatistics/DH_115426
Germany	Paul Ehrlich Institute	http://www.pei.de
Portugal	Portuguese Ministry of Health	http://www.dgs.pt/
Finland	Finnish National Institute for Health and Welfare	http://www.thl.fi/fi_FI/web/fi/sikainfluenssa/sikainfluenssarokote/sikainfluenssarokotusten_kattavuus
Austria	Austria Federal Ministry of Health	http://www.bmg.gv.at/home/Schwerpunkte/Krankheiten/Newsletter_Public_Health/Archiv_2010/
Ireland	Irish Medicines Board	http://www.imb.ie/images/uploaded/documents/IMB_H1N1_Vaccines_Update_1Apr10.pdf
Denmark	State Serum Institute	http://www.ssi.dk/Aktuelt/Nyhedsbreve/EPI-NYT/2010.aspx
Turkey	Turkish Ministry of Health	http://www.grip.gov.tr/
Iceland	Icelandic Directorate of Health	http://www.influensa.is/
Belgium	Interministerial Influenza Coordination Committee	http://www.influenza.be/nl/H1N1_persberichten_nl.asp
Slovenia	Ref. [3]	
Netherlands	National Flu Prevention Program	http://www.snpg.nl/
Switzerland	Federal Office of Public Health	http://www.bag.admin.ch/influenza/

Spain	Spanish Ministry of Health	http://www.aemps.es/profHumana/farmacovigilancia/docs/infoReAdver_SEFV-H/infoFinal_gripeA_H1N1.pdf
Greece	Ref. [2]	
Tunisia	Tunisian Ministry of Health, as reported by “La Presse de Tunisie”	http://fr.allafrica.com/stories/201001140610.html
Czech Republic	Czech Ministry of Health	http://www.pandemie.cz/aktuality/aktualizovane-informace-o-pripadech-potvrzene-nakazy-virem-pandemic-h1n1-2009-329
Norway	Norwegian Institute of Public Health	http://www.fhi.no/

F. Distribution of the simulated peak times in India.

India extends over more than 3 million square kilometers across the Equator and hosts several climatic regions, therefore the influenza patterns across the country are usually very different among regions.

During the 2009 pandemic, India experienced two major waves: a first one, during the summer in Southern and Central India, and a second one, during the winter in Northern India. This resulted in a double peaked profile of the pandemic incidence at national level, as reported by the national Indian surveillance monitoring system.

As displayed in Figure S3, our baseline SFO presents a double peak in the incidence curve of India in more than 90% of the stochastic realizations of the model, with a good agreement with the timing reported by the surveillance.

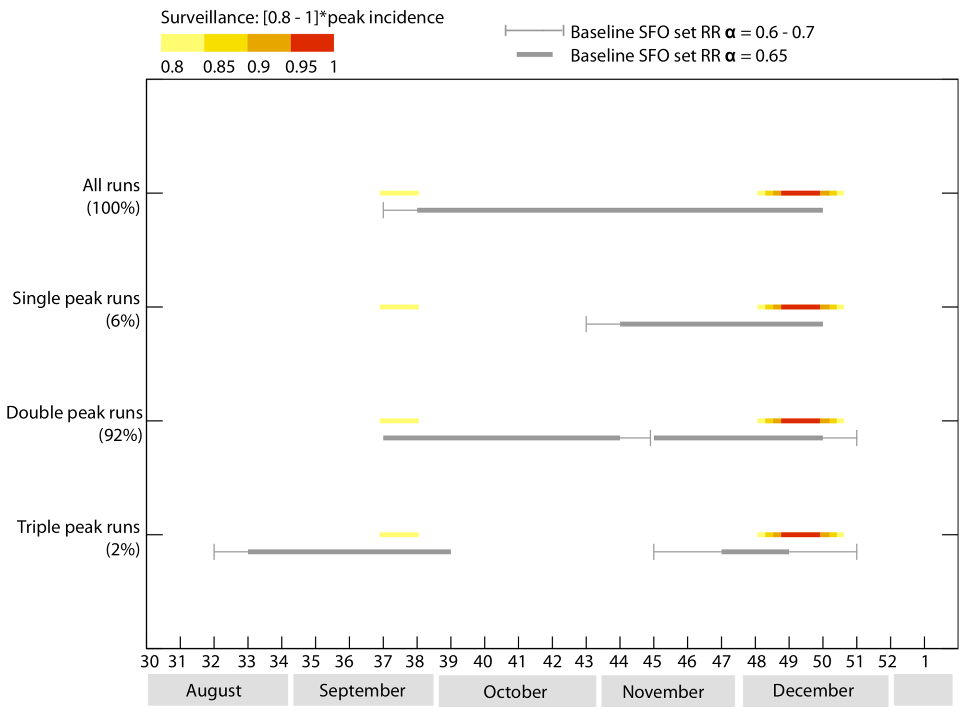

Figure S3. **Simulated peak time in India.** Peak weeks of the epidemic activity in the baseline SFO (grey) for India. The 95% reference ranges of the simulated peak week are obtained by the analysis of 2,000 stochastic realizations of the model for three different values of the seasonal rescaling factor, α_{min} = 0.6, 0.65 and 0.7. Furthermore, the peak week analysis is restricted to three subsets of runs, selected according to the number of peaks of each incidence curve: single peak runs, double peak runs and triple peak runs. The 95% reference ranges are measured on each peak week. For triple peak runs, only the reference ranges of the earliest and the latest peak week are shown. The peak weeks reported by the surveillance are shown as color gradients, whose limits correspond to the time interval where an incidence higher than 80% of the maximum incidence was observed.

G. Regional peaks of the 2009 A/H1N1pdm in Mexico

Similarly to the case of India, Mexico is crossed by the Tropic of Cancer and Mexican regions experienced the pandemic peak at different times between the summer and the winter of 2009, depending on their latitude. The spring pandemic wave in April 2009 mainly affected Mexico City and central states. Mexican southern states experienced a single summer wave, that peaked in the end of June 2009, while central and northern states experienced an early fall wave, that peaked in the end of September 2009 [4].

Our assumption for the seasonal rescaling of influenza transmission considers the central region of Mexico to be in the no-seasonality zone, differently from the rest of the North American countries. This approach effectively takes into account the seasonal aspects induced by the weather conditions [5] and by the yearly calendar of social activities, such as e.g. the school calendar that is found to be strongly related to epidemic onset [6] and influenza transmission [7]. In performing a detailed analysis of Mexico, the similarity between the Mexican school calendar and the one followed in the other countries of North America, points out to the fact that central and northern regions of Mexico are best described by the seasonality of North America, assuming that the transmission potential follows the rescaling function of the Northern Hemisphere. We thus modified the seasonal scaling in those regions assuming that all northern states and central states (highlighted in blue and red in the map of Figure S4) experience seasonality as the countries in the Northern Hemisphere, while we keep the southern states out of the temperate region, without seasonality.

Setting these initial conditions on seasonality, we have calibrated the model using our standard Monte Carlo likelihood procedure and keeping all other conditions equal to the baseline SFO set. We find that estimated values of R_0 and $\alpha_{\min}$ falls within the errors of the baseline SFO set. In particular the estimated values are: R_0 = 1.80 [1.75 – 1.85] and $\alpha_{\min}$ = 0.65 [0.6 – 0.7]. At the global level, the new calibration does not significantly alter the distribution of peak times in the countries of the Northern Hemisphere.

Figure S4 shows the predicted peak times of the model for the three Mexican regions, along with the major peak times observed by the Mexican surveillance, as reported in Ref. [4]. Overall, the model well reproduces the three waves observed in reality, by each region.

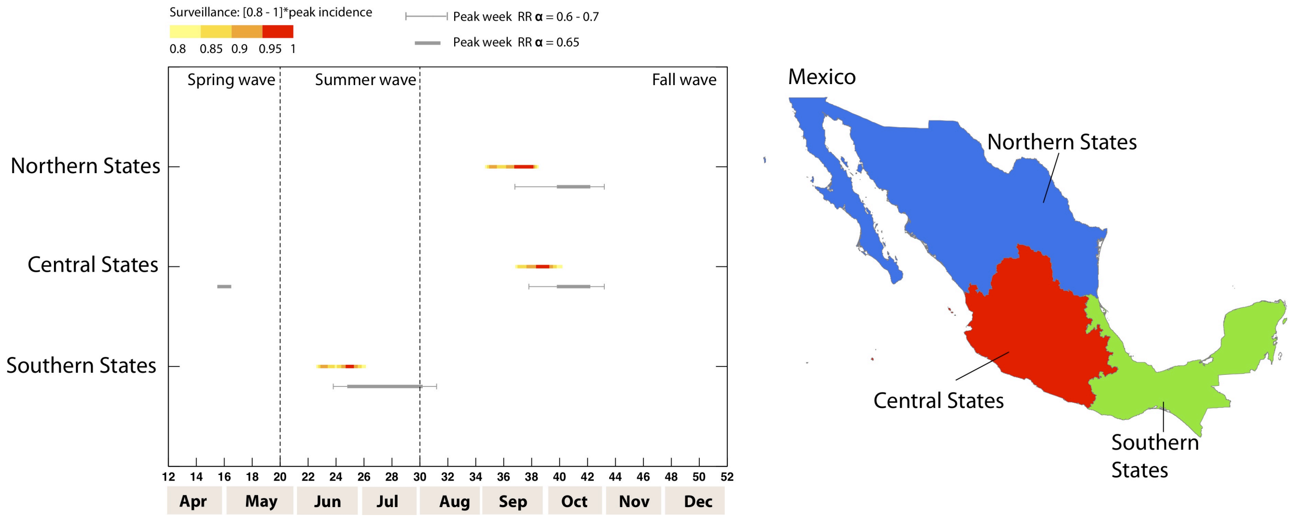

Figure S4. Peak timing in Mexico: simulations and real data. Peak weeks of the epidemic activity for three Mexican regions, in the scenario with seasonality in the northern and central Mexican states. The 95% reference ranges of the simulated peak week are obtained by the analysis of 2,000 stochastic realizations of the model for three different values of the seasonal rescaling factor, α_{min} = 0.6, 0.65 and 0.7. Only in central states the model predicts two peaks: the first peak in April-May and the second peak in September-October. Both reference ranges are provided. The peak weeks reported by the surveillance are shown as color gradients, whose limits correspond to the time interval where an incidence higher than 80% of the maximum incidence was observed.

H. Effect of pre-exposure immunity on pandemic timing

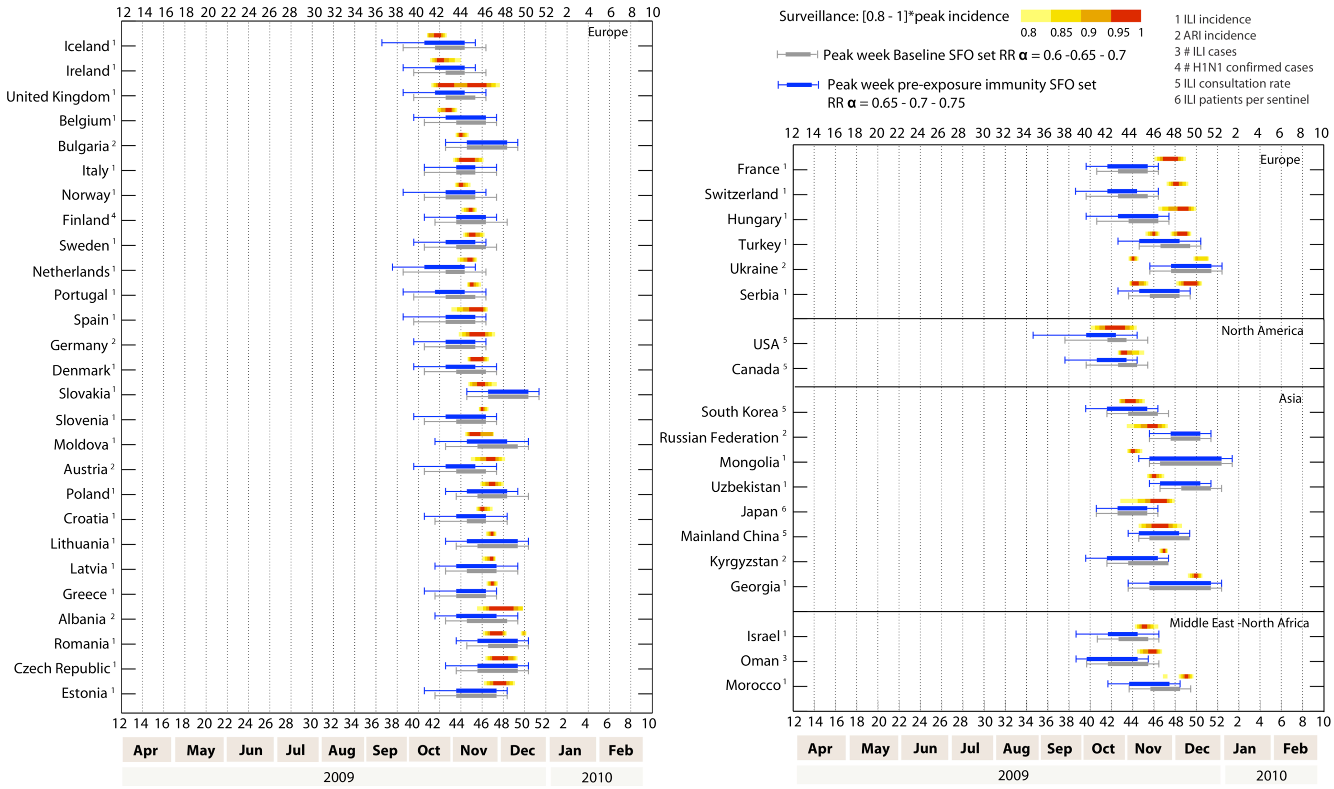

Figure S5. **Peak timing in the Northern Hemisphere: effect of changes in the immunity profile of the population.** Peak weeks of the epidemic activity in the baseline SFO (grey) and in the pre-exposure immunity SFO set (blue). The reference ranges of the baseline SFO peak week are obtained by the analysis of 2,000 stochastic realizations of the model for three different values of the seasonal rescaling factor, α_{min} = 0.6, 0.65 and 0.7. The reference ranges of the pre-exposure SFO peak week are obtained by the analysis of 2,000 stochastic realizations of the model for three different values of the seasonal rescaling factor, α_{min} = 0.65, 0.7 and 0.75.

The peak weeks reported by the surveillance are shown as color gradients, whose limits correspond to the time interval where an incidence higher than 80% of the maximum incidence was observed. Numbers from 1 to 5 indicate the kind of data provided by the surveillance of each country. Numbered weeks of the year correspond to the calendar used by the US Center for Diseases Control and Prevention.

I. Effect of asymptomatic infections on clinical attack rates

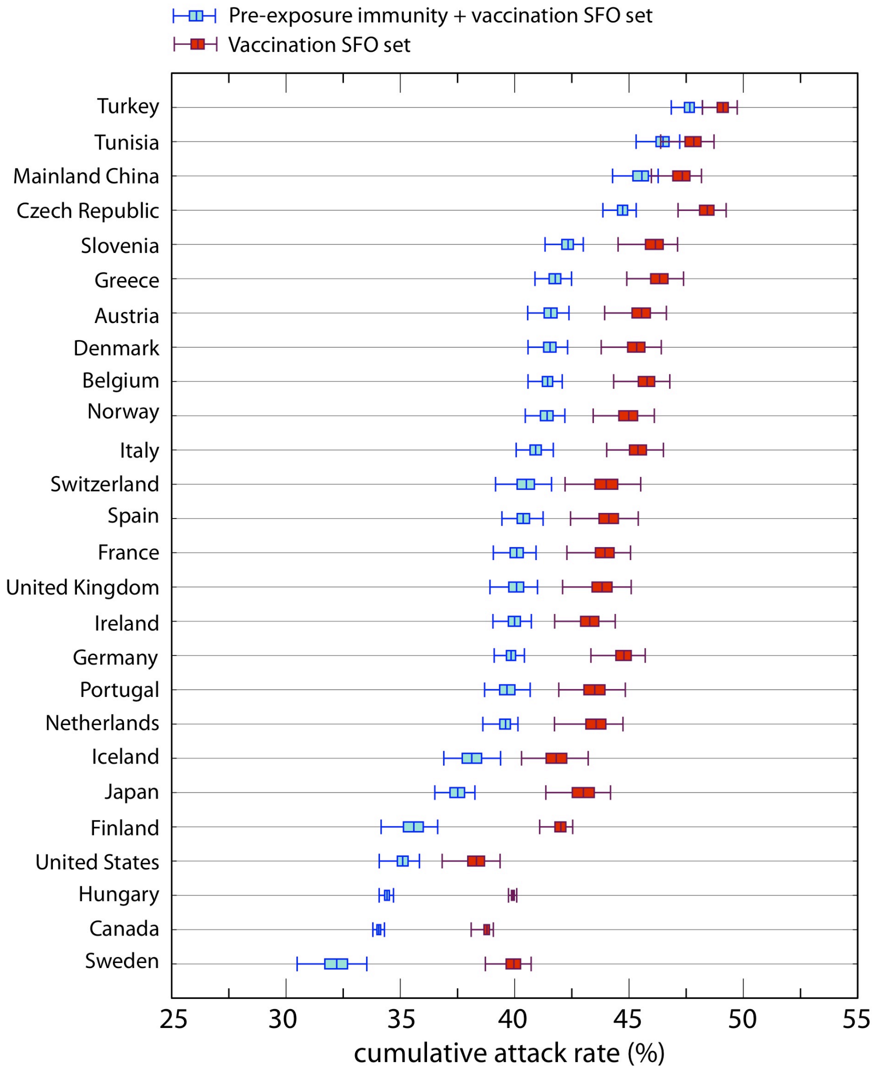

Figure S6. Clinical attack rates with smaller proportion of asymptomatic infections. Clinical attack rate in 26 selected countries of the Northern Hemisphere, for the reference SFO set and pre-exposure immunity SFO set, both with reactive vaccination considered as intervention and a proportion of asymptomatic infections p_a= 33%. The boxplot indicates the 95% and 50% reference ranges, with the median value, of the simulated attack rates obtained by the analysis of 2,000 stochastic realizations of the model for α_{min} = 0.65.

J. Sensitivity analysis on the minimum seasonal rescaling factor

If we assume a minimum value of the seasonal rescaling factor $\alpha_{min} = 0.1$, as in the case of seasonal influenza [8], simulations would predict a late activity peak, with respect to our baseline scenario. Figure S7 displays the peak week reference ranges for the two scenarios in 46 countries of the Northern Hemisphere. Setting $\alpha_{min} = 0.1$ results in a delay of about 1.5 months for all countries.

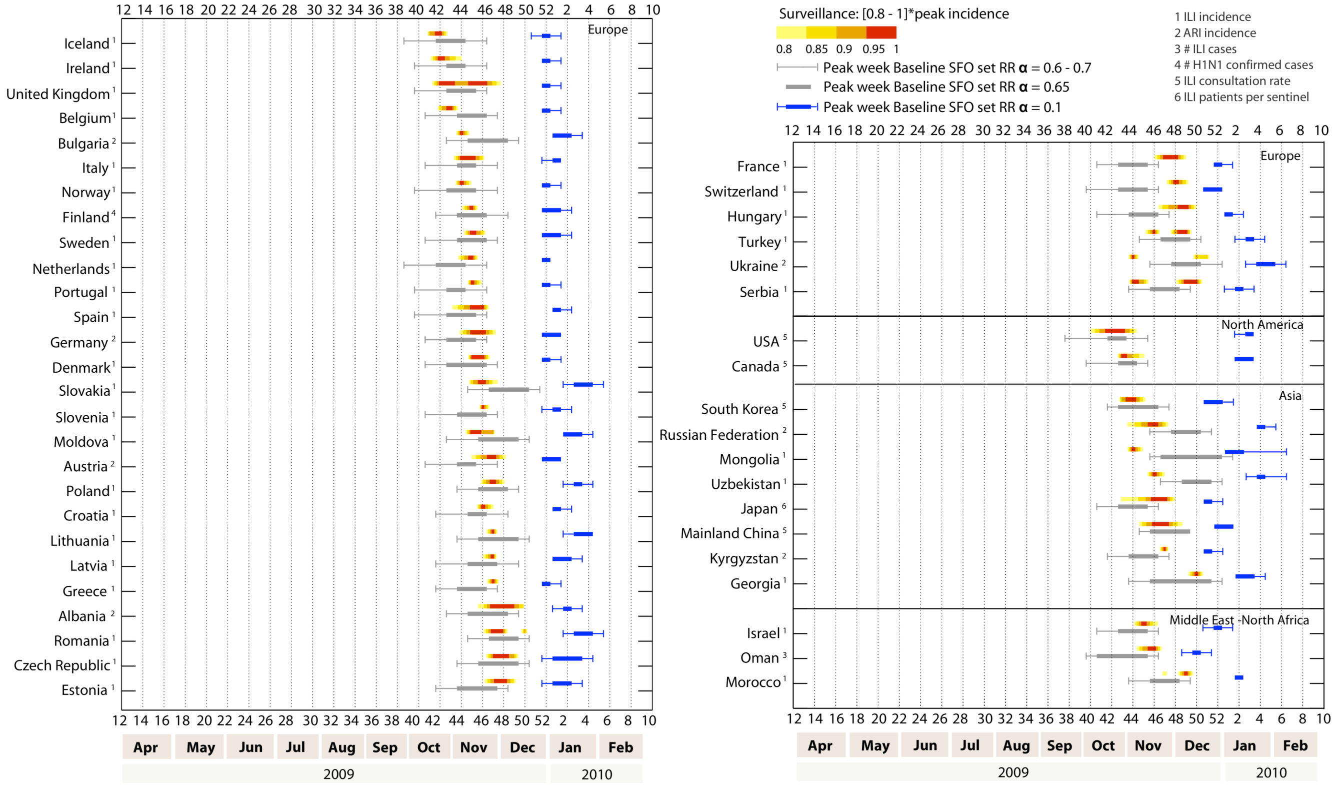

Figure S7. **Peak timing in the Northern Hemisphere: effect of changes in the minimum seasonal rescaling.** Peak weeks of the epidemic activity in the baseline SFO set (grey) and in the $\alpha_{min} = 0.1$ scenario (blue). The reference ranges of the baseline SFO set are obtained by the analysis of 2,000 stochastic realizations of the model for three different values of the seasonal rescaling factor, α_{min} = 0.6, 0.65 and 0.7. The peak weeks reported by the surveillance are shown as color gradients, whose limits correspond to the time interval where an incidence higher than 80% of the maximum incidence was observed. Numbers from 1 to 5 indicate the kind of data provided by the surveillance of each country. Numbered weeks of the year correspond to the calendar used by the US Center for Diseases Control and Prevention.

K. Sensitivity analysis on the relative infectiousness of asymptomatic individuals

We explore the role of the relative infectiousness of asymptomatic individuals in the pre-exposure immunity SFO, with a proportion of asymptomatic infections p_a = 45% and assuming r_β = 0.1. Results are almost unaffected by this change in the parameter values, as shown in Figure S8.

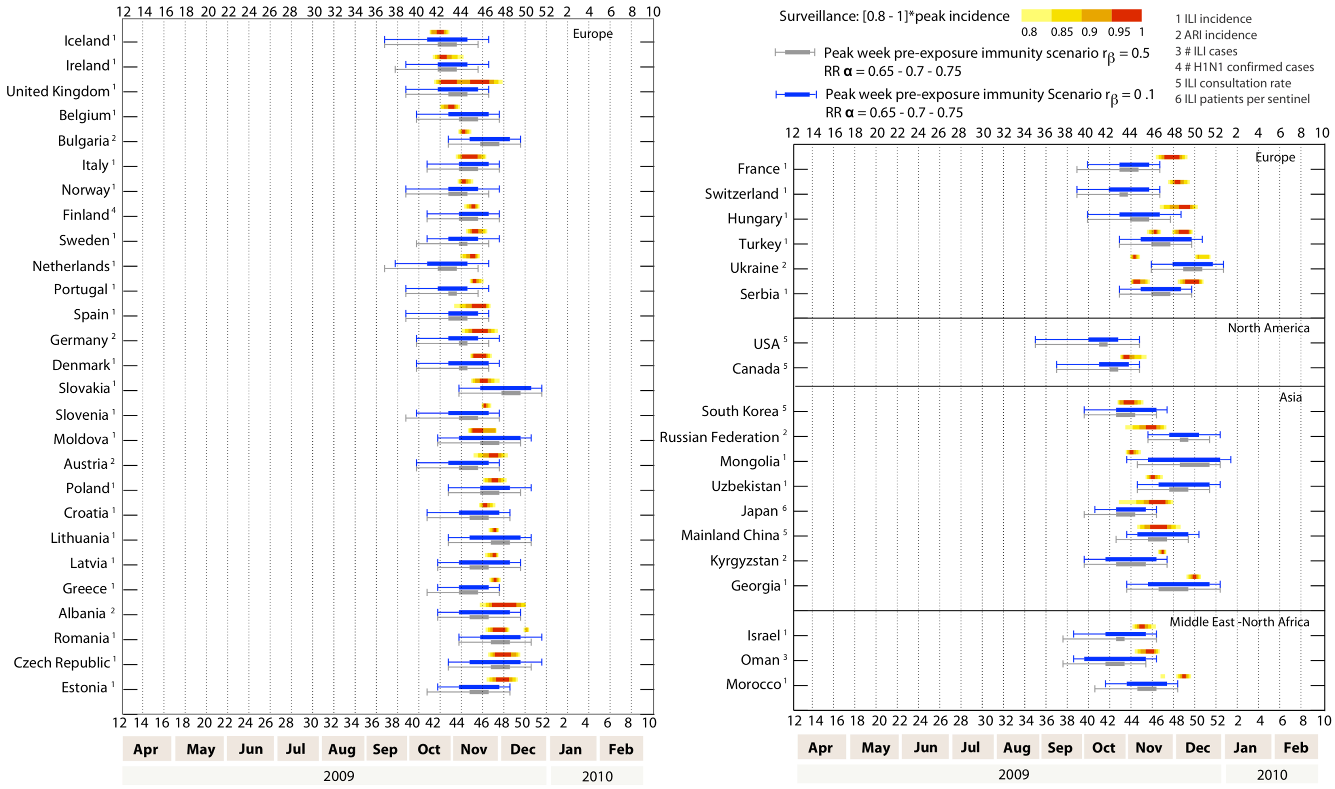

Figure S8. **Peak timing in the Northern Hemisphere: effect of changes in the relative infectiousness of asymptomatic individuals**. Peak weeks of the epidemic activity in the pre-exposure immunity SFO with r_β = 0.5 (grey) and with r_β = 0.1 (blue). The reference ranges of the simulated peak week are obtained by the analysis of 2,000 stochastic realizations of the model for three different values of the seasonal rescaling factor, α_{min} = 0.65, 0.7 and 0.75. The peak weeks reported by the surveillance are shown as color gradients, whose limits correspond to the time interval where an incidence higher than 80% of the maximum incidence was observed. Numbers from 1 to 5 indicate the kind of data provided by the surveillance of each country. Numbered weeks of the year correspond to the calendar used by the US Center for Diseases Control and Prevention.

L. Sensitivity analysis on the initial conditions and changes in the seed location

The identification of the initial conditions of the epidemic is an example in which both data knowledge and model resolution can induce possible inaccuracies. This could be due to partial information available on the initial seed during the early stage of the outbreak, or also, given complete data knowledge, on the lack of high enough resolution to correctly model the seed. Alternative solutions based on the structural data integrated in the model have to be therefore devised. Here we explore the sensitivity of the simulation results with respect to changes in the geographic location of the initial conditions of the outbreak, assuming the seed to be Mexico City, instead of the town of the census area of La Gloria, as was done in Ref. [9]. We perform a new calibration of the model and find a lower estimate of the reproductive number, R_0=1.5, and no signal for seasonal effects. We assume therefore that in all regions of the world the reproductive number is fixed in time and corresponds to its estimated value. Figure S9 shows the difference between the median peak days in the scenario with the seed location in Mexico City, and the reference SFO set, where the seed is located near La Gloria; only the 500 busiest airports of the database are shown in the plot for reference. As a consequence of the low transmissibility and the absence of seasonal rescaling the pandemic activity peaks were radically anticipated in the Northern Hemisphere and postponed in the Tropics and Southern Hemisphere, with respect to the reference SFO set. In the countries of the Northern Hemisphere, the shift was generally larger than 4 weeks and extended up to 11 weeks, corresponding to a very early incidence peak between August and September. These results were largely far from the observed epidemic patterns of the 2009 – 2010 winter season, showing that inaccuracies in the seed location (due to limited data availability on the outbreak or to limited resolution scale of the model) could greatly alter the results.

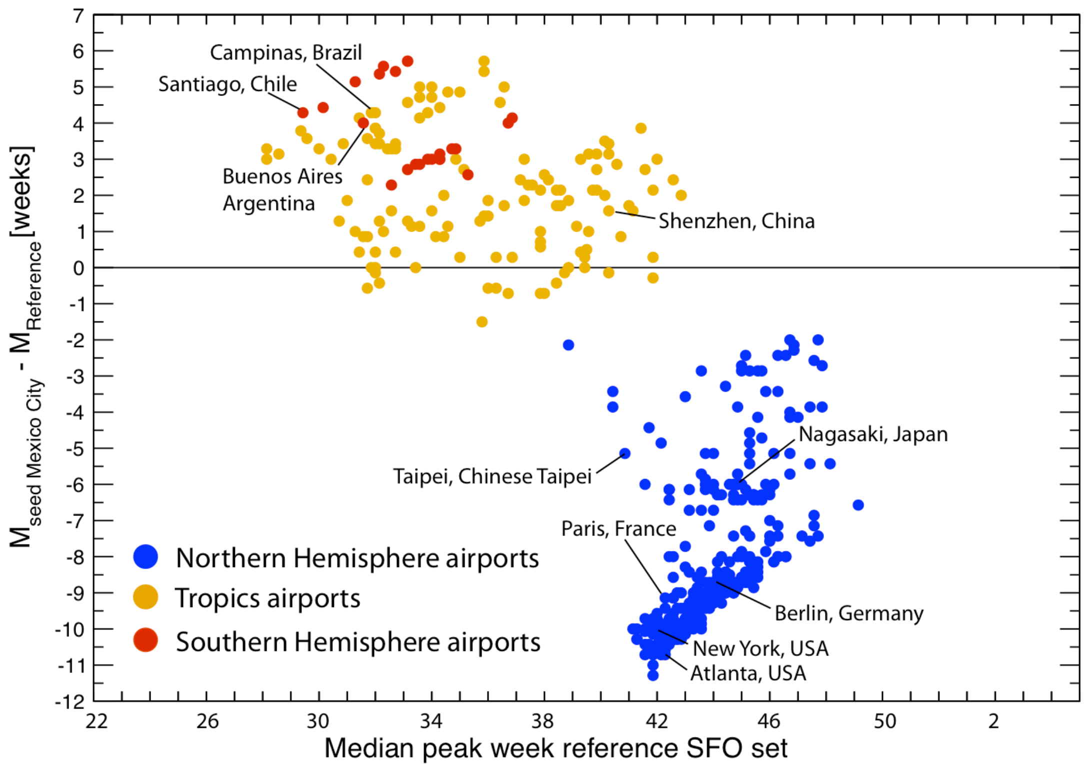

Figure S9. Peak timing: effect of changes in the seed location. Difference of the median peak weeks in the reference SFO set, with initial seed in La Gloria, and the scenario with initial seed in Mexico City, for the 500 busiest airports, as a function of the median peak week in the reference SFO set. Dots are color coded according to the corresponding airport's climate zone.

References

1. Balcan D, Colizza V, Gonçalves B, Hu H, Ramasco JJ, Vespignani A: **Multiscale mobility networks and the large scale spreading of infectious diseases.** Proc. Natl. Acad. Sci. USA 2009, **106**: 21484-21489.
2. Tsiodras S, Sypsa V, Hatzakis A: **The vaccination campaign and against 2009 pandemic influenza A(H1N1) and its continued importance in view of the uncertainty surrounding the risk associated with the pandemic.** Euro Surveill. 2010, **15**:3.
3. Kraigher A, Učakar V: **Surveillance of adverse events following immunization against pandemic influenza in Slovenia in season 2009/10.** Vaccine 2010, **28**:5467 – 5472.
4. Chowell G, Echevarria-Zuno S, Viboud C, Simonsen L, Tamerius J, Miller MA, Borja-Aburto, VH: **Characterizing the epidemiology of the 2009 H1N1 influenza A/H1N1 pandemic in Mexico.** PLoS Med. 2011, **8(5)**: e1000436.

5. Chao DL, Halloran ME, Longini Jr IM: **School opening dates predict pandemic influenza A (H1N1) epidemics in the United States.** J. Infect. Dis. 2010, **202(6)**: 877-880.
6. Shaman J, Goldstein E, Lipsitch M. **Absolute humidity and pandemic versus epidemic influenza**. Am. J. Epidemiol. 2011, **173(2):**127.
7. Cauchemez S et al. **Closure of schools during an influenza pandemic**. Lancet Infect. Dis. 2009, **9(8)**: 473.
8. Cooper BS, Pitman RJ, Edmunds WJ, Gay N: **Delaying the international spread of pandemic influenza.** PLoS Med. 2006, **3**:e12.
9. Flahault A, Vergu E, Boelle P-Y: **Potential for a global dynamic of Influenza A (H1N1).** BMC Infect. Dis. 2009, **9**:129.
